# A Novel Cargo Delivery System‐AnCar‐Exo^LaIMTS^ Ameliorates Arthritis via Specifically Targeting Pro‐Inflammatory Macrophages

**DOI:** 10.1002/advs.202306143

**Published:** 2023-12-11

**Authors:** Song Li, Ya‐ran Wu, Xiu‐qin Peng, Han‐gang Chen, Tong‐yi Zhang, Hua Chen, Jing Yang, Yang‐li Xie, Hua‐bing Qi, Wei Xiang, Bo Huang, Si‐ru Zhou, Yan Hu, Qiao‐yan Tan, Xiao‐lan Du, Jun‐lan Huang, Ruo‐bin Zhang, Xiao‐hong Li, Feng‐tao Luo, Min Jin, Nan Su, Xiao‐qing Luo, Shuo Huang, Peng Yang, Xiao‐Jing Yan, Ji‐qin Lian, Ying Zhu, Yan Xiong, Gong‐yi Xiao, Ying‐ying Liu, Chen Shen, Liang Kuang, Zhen‐hong Ni, Lin Chen

**Affiliations:** ^1^ Center of Bone Metabolism and repair laboratory for Prevention and rehabilitation of Training injuries State Key laboratory of Trauma Burns and combined injury Trauma center Research Institute of Surgery Daping Hospital Army Medical University (Third Military Medical University) Chongqing 400000 China; ^2^ Department of Clinical Biochemistry Faculty of Pharmacy and Laboratory Medicine Army Medical University (Third Military Medical University) Chongqing 40000 China; ^3^ State Key Laboratory of Trauma Burns and Combined Injury Department of Rehabilitation Medicine Daping Hospital Army Medical University (Third Military Medical University) Chongqing 400000 China; ^4^ Department of Military Basic Training and Army Management Army Health Service Training Base Army Medical University (Third Military Medical University) Chongqing 400000 China; ^5^ Department of Biochemistry and Molecular Biology College of Basic Medical Sciences Army Medical University (Third Military Medical University) Chongqing 400000 China; ^6^ Department of Rehabilitation Medicine The First Affiliated Hospital of Chongqing Medical University Chongqing 400000 China; ^7^ Center for Joint Surgery Department of Orthopedic Surgery The Second Affiliated Hospital of Chongqing Medical University Chongqing 400000 China

**Keywords:** arthritis, exosome, HIF‐1α

## Abstract

Macrophages are heterogenic phagocytic cells that play distinct roles in physiological and pathological processes. Targeting different types of macrophages has shown potent therapeutic effects in many diseases. Although many approaches are developed to target anti‐inflammatory macrophages, there are few researches on targeting pro‐inflammatory macrophages, which is partially attributed to their non‐s pecificity phagocytosis of extracellular substances. In this study, a novel recombinant protein is constructed that can be anchored on an exosome membrane with the purpose of targeting pro‐inflammatory macrophages via antigen recognition, which is named AnCar‐Exo^LaIMTS^. The data indicate that the phagocytosis efficiencies of pro‐inflammatory macrophages for different AnCar‐Exo^LaIMTS^ show obvious differences. The AnCar‐Exo^LaIMTS3^ has the best targeting ability for pro‐inflammatory macrophages in vitro and in vivo. Mechanically, AnCar‐Exo^LaIMTS3^ can specifically recognize the leucine‐rich repeat domain of the TLR4 receptor, and then enter into pro‐inflammatory macrophages via the TLR4‐mediated receptor endocytosis pathway. Moreover, AnCar‐Exo^LaIMTS3^ can efficiently deliver therapeutic cargo to pro‐inflammatory macrophages and inhibit the synovial inflammatory response via downregulation of HIF‐1α level, thus ameliorating the severity of arthritis in vivo. Collectively, the work established a novel gene/drug delivery system that can specifically target pro‐inflammatory macrophages, which may be beneficial for the treatments of arthritis and other inflammatory diseases.

## Graphical abstract

Targeting different types of macrophages has shown potent therapeutic effects in arthritis. This study constructs new engineered exosomes that can efficiently deliver therapeutic contents to pro‐inflammatory macrophages mainly through TLR4‐mediated endocytosis, thereby ameliorating the severity of arthritis in vivo. This work provides a novel prospective and potential strategy for targeting treatments of arthritis and other inflammatory diseases.

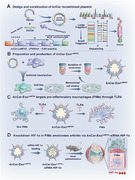

## 1 Introduction

Macrophages play a crucial role in innate immune responses, contributing to many physiological and pathological processes including tissue injuries and repair.^[^ ^1^ ^]^ Following injury, pro‐inflammatory macrophages (classically activated macrophages) begin to infiltrate in injured tissue, contributing to clearing the damaged cells and bacteria. Subsequently, the damaged tissue will go through a repairing process accompanied by anti‐inflammatory macrophages (alternatively activated macrophages). The imbalance between pro‐inflammatory and anti‐inflammatory macrophages will lead to dysregulated tissue repair or even chronic inflammation.^[^ ^2^ ^]^ Targeting different types of macrophages has shown potent therapeutic effects in multiple diseases. Although multiple approaches have been developed to target anti‐inflammatory macrophages, there are few researches on targeting pro‐inflammatory macrophages partially because of their non‐specificity phagocytosis.

Synovial macrophages are the major immune cells in joint microenvironment that actively participate in the repairing of articular injuries. Previous studies have demonstrated that there are a large amount of pro‐inflammatory macrophages in inflammatory arthritis, which play important roles in the pathological damage of joints by secreting a variety of cytokines.^[^ ^3^ ^]^ Liu et al. found that the ratio of pro‐inflammatory macrophages to anti‐inflammatory macrophages in synovial fluid was higher in inflammatory arthritis patients compared to the health group.^[^ ^4^ ^]^ Meanwhile, the inflammatory cytokines secreted by pro‐inflammatory macrophages, IL‐1β and TNF‐α, could promote osteoclastogenesis, stimulating the secretion of MMPs and cytokines.^[^ ^5^ ^]^ In contrast, inhibiting the hyperactivation of pro‐inflammatory macrophages or raising the ratio of anti‐inflammatory to pro‐inflammatory macrophages could remarkably relieve the symptoms of inflammatory arthritis and ameliorate progress of disease.^[^ ^6^ ^]^ However, the current treatment targeting synovial pro‐inflammatory macrophages lacks specificity and has potential off‐target impacts on other types of articular cells including chondrocytes, synovial fibroblasts, anti‐inflammatory macrophages and endotheliocytes. Therefore, it is necessary to develop novel drug delivery systems to specifically target synovial pro‐inflammatory macrophages, aiming to improve the therapeutic effect of anti‐inflammatory molecules against arthritis.

Exosomes are membrane‐bound vehicles with sizes ranging from 30 to 150 nm, which were released from cells through membrane fusion between multivesicular bodies and the plasma membrane.^[^ ^7^ ^]^ As a carrier of DNA, RNA and protein, exosome functions as mediator for intercellular communication and substance exchange.^[^ ^8^ ^]^ Due to its excellent immune compatibility and high organotropism, exosome is regarded as a novel potent drug delivery platform for the treatment of many diseases. In recent years, there have been increasing studies focusing on the targeting ability of exosomes for specific types of cells. Kanuma et al. and Wood et al. have modified N segment of exosome membrane protein CD163 or LAMP2 by expression‐specific antigen sequences, which mediates the uptake of engineering exosomes by targeted cells.^[^ ^9^ ^]^ For macrophages, Gowri et al. reported that exosomes with expression of IL‐4 receptor on their membrane inhibit tumor growth by targeting tumor‐associated macrophages.^[^ ^10^ ^]^ Furthermore, the exosomes, which were engineered to express molecules on exosome membrane targeting scavenger receptor class A, can ameliorate joint inflammation.^[^ ^11^ ^]^ Although these engineered exosomes have exhibited certain therapeutic effects, they also have several limitations including complex preparation process, presence of potentially harmful chemical species and lack of targeting specificity for subset populations of macrophages, especially pro‐inflammatory macrophages.^[^ ^12^ ^]^ Therefore, novel delivery methods targeting pro‐inflammatory macrophages based on exosomes are needed to enhance the therapeutic effects for arthritis.

**Figure 1:**
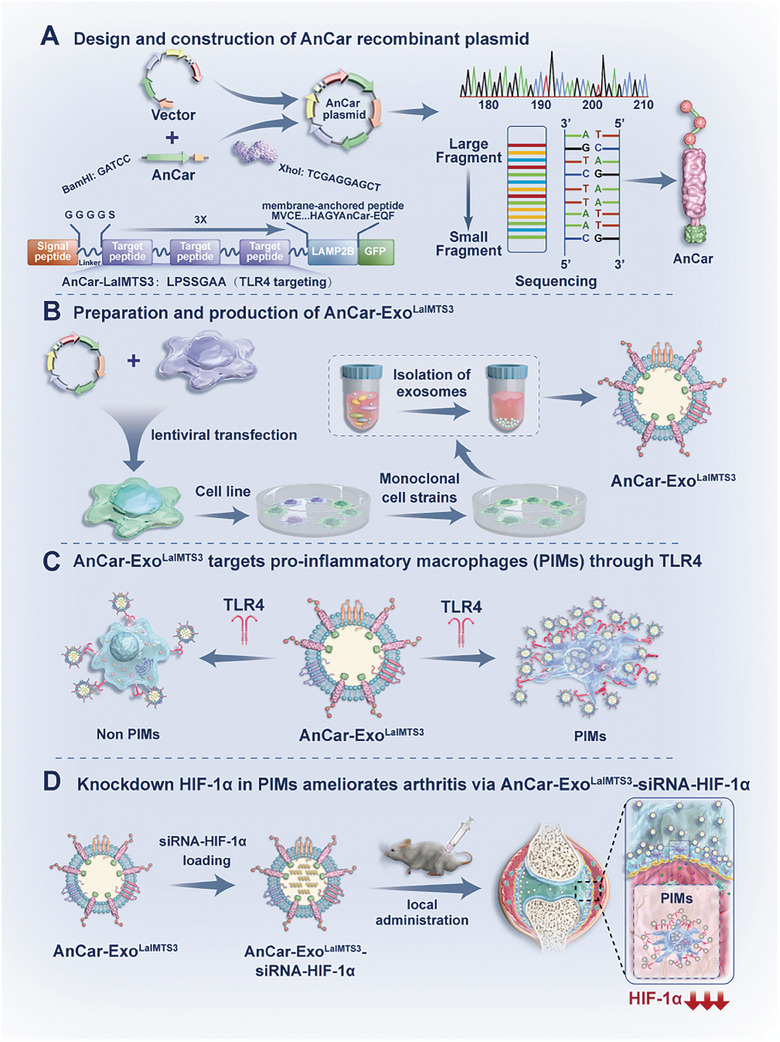
Schematic illustration of AnCar‐Exo^LaIMTS^ and the potential application for inflammatory arthritis. A) the design and construction of AnCar recombinant plasmid. The AnCar is composed of four elements, including exosome membrane anchoring sequence, pro‐inflammatory macrophage targeting sequence, exosome tracing sequence, flexible linker region sequence. B) Preparation and production of AnCar‐Exo^LaIMTS3^. C) AnCar‐Exo^LaIMTS3^ targets pro‐inflammatory macrophages (PIMs) through TLR4. D) Knockdown HIF‐1α in PIMs ameliorates arthritis via AnCar‐Exo^LaIMTS3^‐siRNA‐HIF‐1α. AnCar‐Exo^LaIMTS3^‐siRNA‐HIF‐1α can efficiently inhibit the expression of HIF‐1α in synovial macrophages, decrease synovial inflammatory response, ameliorate gait abnormalities and pathological change in mice arthritis model.

Here, we construct a novel recombinant protein that could be anchored on exosome membranes with the purpose of targeting pro‐inflammatory macrophages. Just like the Chimeric Antigen Receptor (CAR), this recombination protein consists of four major components including 1) N‐terminal antigen domain that recognizes Toll‐like Receptor 4 (TLR4), 2) flexible linker region, 3) transmembrane region of LAMP2B, 4) C‐terminal GFP. Moreover, this recombination protein would help exosomes to specifically recognize and regulate the targeted cells (pro‐inflammatory macrophages), which is similar to the function of CAR in engineered cells. Different from the CAR, this recombinant protein is mainly located on the bilayer membrane of exosome and does not have the function of signal transduction. Based on the above, we named this recombinant protein as AnCar‐LaIMTS (analogous chimeric antigen pseudo‐receptor with LAMP2B sequence that targets pro‐inflammatory macrophages). In this study, AnCar‐LaIMTS is used to modify exosomes deriving from seeding cells with purpose of delivering cargo specifically to pro‐inflammatory macrophages, which is named AnCar‐Exo^LaIMTS^. Our data indicate that the phagocytosis efficiencies of pro‐inflammatory macrophages for different AnCar‐Exo^LaIMTS^ show obvious differences. In the existing candidate range, the AnCar‐Exo^LaIMTS3^ has the best targeting ability to pro‐inflammatory macrophages in vitro and in vivo. Moreover, our bioinformatics analysis revealed the existence of the AnCar‐LaIMTS3 amino acid sequence within the Leucine‐rich repeat flightless‐interacting protein 1, which is recognized for its interaction with the Leucine‐rich repeat domain located in the extracellular portion of TLR4. Through the application of TLR4 neutralizing antibodies or by employing TLR4^−/−^ mice, we found that this high‐efficiency targeting of pro‐inflammatory macrophages is achieved through TLR4‐mediated endocytosis. In addition, AnCar‐Exo^LaIMTS3^ loading with HIF‐1α‐siRNA (AnCar‐Exo^LaIMTS3^‐siRNA‐HIF‐1α) could efficiently deliver cargo (HIF‐1α‐siRNA) into pro‐inflammatory macrophages. Through intra‐articular administration of AnCar‐Exo^LaIMTS^‐siRNA‐HIF‐1α, the expression of HIF‐1α in pro‐inflammatory macrophages of synovium is significantly downregulated, while the level of HIF‐1α is rarely influenced in other types of cells. On the contrary, the exosomes carrying HIF‐1α‐siRNA without AnCar‐LaIMTS randomly enter into multiple types of cells with limited interference effects on HIF‐1α in pro‐inflammatory macrophages of synovium (**Figure** **1**). In brief, we first found that pro‐inflammatory macrophages have selective endocytosis on engineered exosomes modified by different AnCar‐LaIMTS. AnCar‐Exo^LaIMTS3^ can improve the targeting ability of the pro‐inflammatory macrophages and promote the efficient enrichment of therapeutic cargo in target cells through TLR4. We believe that this novel delivery system of AnCar‐Exo^LaIMTS^ will be beneficial for the treatment of arthritis or other inflammatory diseases.

## 2 Results and Discussion

### 2.1 Design and Construction of AnCar‐LaIMTS Plasmids

**Figure 2:**
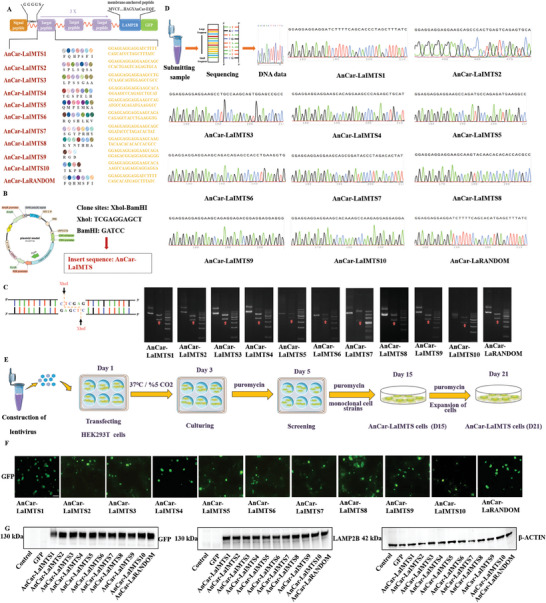
Design and construction of AnCar‐LaIMTS plasmids. A) The major components of AnCar‐LaIMTS consisted of four elements. B) Eleven kinds of AnCar‐LaIMTS sequences were inserted into PLVX‐puro plasmids. C) The results of electrophoresis images. Target genes were digested at XhoI and BamHI sites. AnCar‐LaIMTS sequence was labelled by red arrow. D) DNA sequencing analysis. Sequences of DNA were consistent with peptide sequences. E) Eleven kinds of AnCar‐LaIMTS plasmids were transfected into HEK 293T cell through lentivirus. F) GFP protein expression in 11 cell lines. The expressed GFP‐fusing proteins could be observed green florescence under fluorescent microscopy. Scale bars, 25 µm. G) Western Blot analysis of fusion protein in cells. Antibodies were anti‐LAMP2B and anti‐GFP. Control means HEK 293T. GFP means HEK 293T transfected with blank GFP protein plasmid.

The major components of AnCar‐LaIMTS were composed of four elements (**Figure** **2**A), including element 1 (exosome membrane anchoring sequence), element 2 (pro‐inflammatory macrophage targeting sequence), element 3 (exosome tracing sequence), element 4 (flexible linker region sequence). For element 1, the sequence coding LAMP2B was used to help AnCar‐LaIMTS anchor on exosome membrane.^[^ ^13^ ^]^ For element 2, sequences derived from a phage display library, which were proposed to recognize pro‐inflammatory macrophages in previous study(29–32), were fused with the N terminus of LAMP2B with flexible linker (element 4). Moreover, GFP coding sequence in element 3 was fused with exosomes’ intracellular segment (C terminus of LAMP2B) so as to track exosomes through GFP fluorescence. To screen and identify the optimal AnCar, ten targeting sequences were used to code element 2, which came out with 10 different AnCar‐LaIMTSs named as AnCar‐LaIMTS1 to AnCar‐LaIMTS10, while the random sequence in element 2 was named as AnCar‐LaRANDOM. Subsequently, all AnCar‐LaIMTSs’ coding sequences were inserted into PLVX‐puro plasmids for lentivirus production (Figure 2B). To test the accuracy of plasmid construction, the eleven plasmids were digested at XhoI and BamHI sites. The electrophoresis results showed that all plasmids produced a fragment of ≈1200 bp, which was consistent with the design size (Figure 2C). The sequences of element 2 of all AnCar‐LaIMTSs were further identified by PCR product sequencing as shown in Figure 2D. These data revealed that the constructed plasmids encoded the exact targeting peptides as designed. Overall, we have successfully designed and constructed 11 plasmids that could efficiently encode 11 different AnCar‐LaIMTS recombination proteins (AnCar‐LaIMTS1∼AnCar‐LaIMTS10 and AnCar‐LaIMTSRANDOM).

Subsequently, these recombinant plasmids were individually transfected into HEK 293T cells through lentivirus to acquire respective cell lines (Figure 2E). As shown in Figure 2F, the GFP green signals of eleven stable cell lines can be observed under fluorescent microscopy. Moreover, we detected the expression of fusion protein by Western Blot. Data showed that both LAMP2B antibody and GFP antibody could detect the expected fusing protein (130 kDa protein band), while HEK 293T cells and HEK 293T cells transfected with GFP plasmid didn't show fusing protein band, demonstrating that AnCar‐LaIMTS proteins could be successfully synthesized in cells (Figure 2G). Collectively, we constructed 11 stable cell lines that could express the corresponding recombinant proteins (AnCar‐ LaIMTS1 to AnCar‐LaIMTS10, AnCar‐ LaRANDOM).

### 2.2 Characteristics of AnCar‐LaIMTS Modified Exosomes

To obtain the corresponding AnCar‐modified exosomes from the above 11 stable cell lines (**Figure** **3**A), Ultracentrifugation was used to isolate exosomes from supernatant as reported previously.^[^ ^7^ ^]^ In brief, the separated supernatants were centrifuged at 300, 2000, and 10 000 g for 10 min. Later, they were centrifuged at 1 00 000 g for 90 min after being filtered through a 0.22 µm filter. Finally, the exosomes were collected and named as AnCar‐Exo^LaCTRL^, AnCar‐Exo^LaRANDOM^, and AnCar‐Exo^LaIMTS1^ to AnCar‐Exo^LaIMTS10^ (Figure 3B). To confirm the success of isolating exosomes from supernatant, aforementioned exosomes were subjected to NanoSight detection. As shown in Figure 3C–F, eleven kinds of AnCar‐modified exosomes as well as AnCar‐Exo^LaCTRL^ presented classic characteristics of exosomes, such as size distribution and exosome structure. The number of exosomes with the diameters between 30 and 200 nm ranged from 1.23 × 10^11^ to 1.92 × 10^11^ particles/ml, and the main particle sizes of exosomes were within 99.8 nm–158 nm with average particle size between 123.6 nm –182.1 nm (Figure 3D), suggesting that there were no significant differences in diameters between the AnCar‐modified exosomes and native exosomes. Besides, we observed exosome‐like structures under transmission electron microscope. It could be seen from Figure 3D that the separated exosome‐like vesicles from supernatant displayed typical saucer‐bilayer membrane, which is a characteristic of exosomes. Moreover, the fusion protein AnCar and surface markers of exosomes including CD63, CD81, and TSG101 could be detected in exosomes (Figure 3E). As shown in Figure 3F, GFP was successfully loaded into AnCar‐Exo^LaRANDOM^, AnCar‐Exo^LaIMTS1^ and AnCar‐Exo^LaIMTS10^ as detected by ImageStreamX compared with AnCar‐Exo^LaCTRL^. Taken together, AnCar‐modified exosomes have been successfully separated from 11 engineered stable cell lines by ultracentrifugation with classic characteristics of exosomes including size, structure and contents.

Figure 3Characteristics of AnCar‐LaIMTS modified exosomes. A) The model and structure of AnCar‐Exo^LaIMTS^. LAMP2B is fused with pro‐inflammatory macrophage sequence peptide. B) A schematic diagram illustrating isolation of AnCar‐Exo^LaCTRL^, AnCar‐Exo^LaIMTS^ and AnCar‐Exo^LaRANDOM^. AnCar‐Exo^LaCTRL^ was derived from HEK 293T cells. C) Particle size distribution of AnCar^LaCTRL^, AnCar‐Exo^LaIMTS^ and AnCar‐Exo^LaRANDOM^ detected by Nanosight. D) The morphology of AnCar^LaCTRL^, AnCar‐Exo^LaIMTS^ and AnCar‐Exo^LaRANDOM^ measured by transmission electron microscope. Scale bars, 100 nm. E) Western Blot analysis of fusion protein in exosomes. Antibodies were anti‐CD63, anti‐CD81, anti‐TSG101 and anti‐LAMP2B. F) GFP fluorescence in exosomes detection by ImageStreamX.

**Figure:**
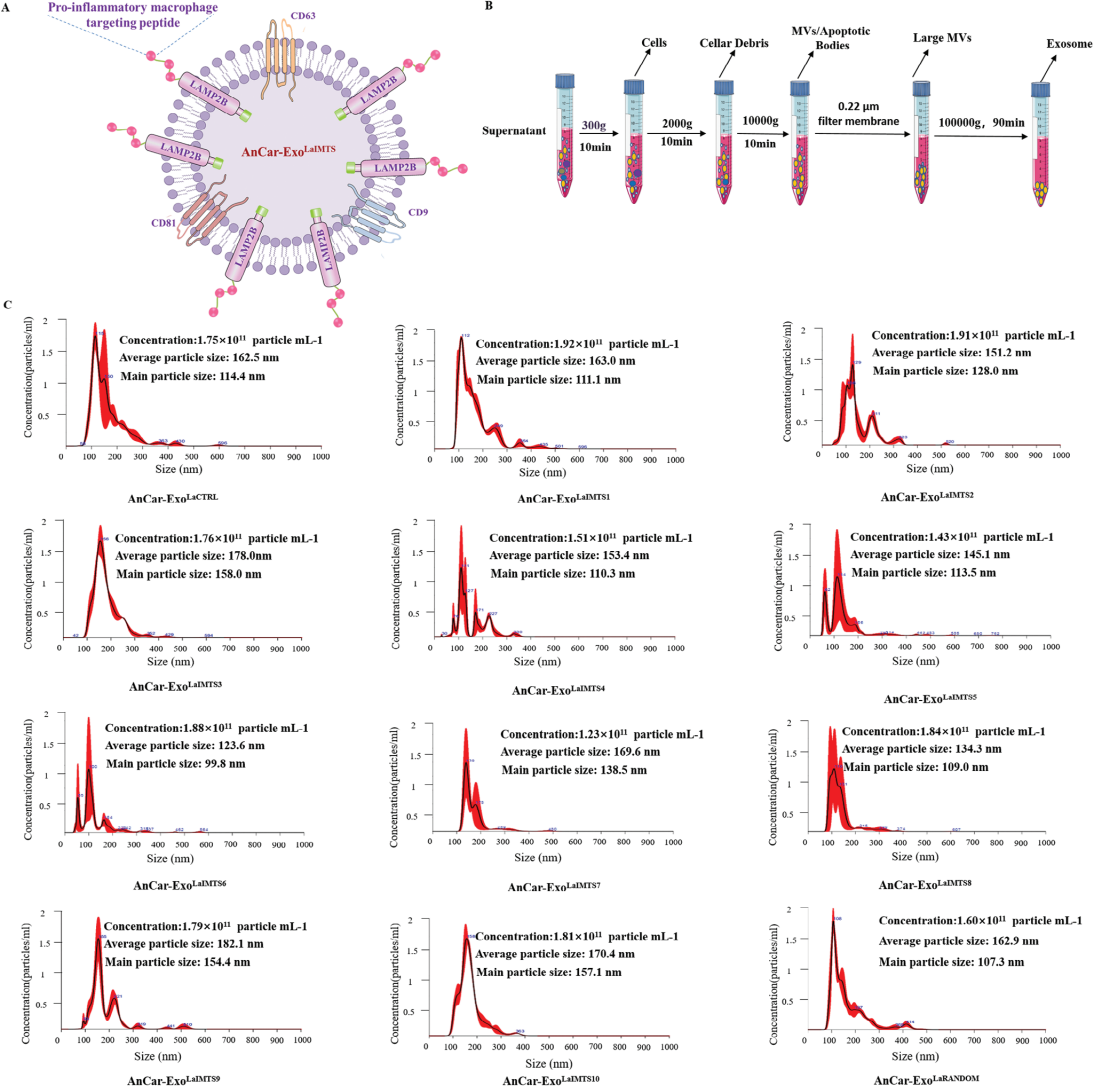

**Figure:**
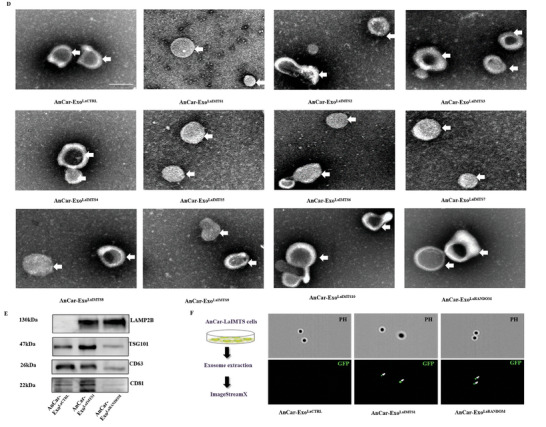

### 2.3 Screening and Identification of the Optimal AnCar‐Exo^LaIMTS^ Specifically Targeting Pro‐Inflammatory Macrophages In Vitro

**Figure 4:**
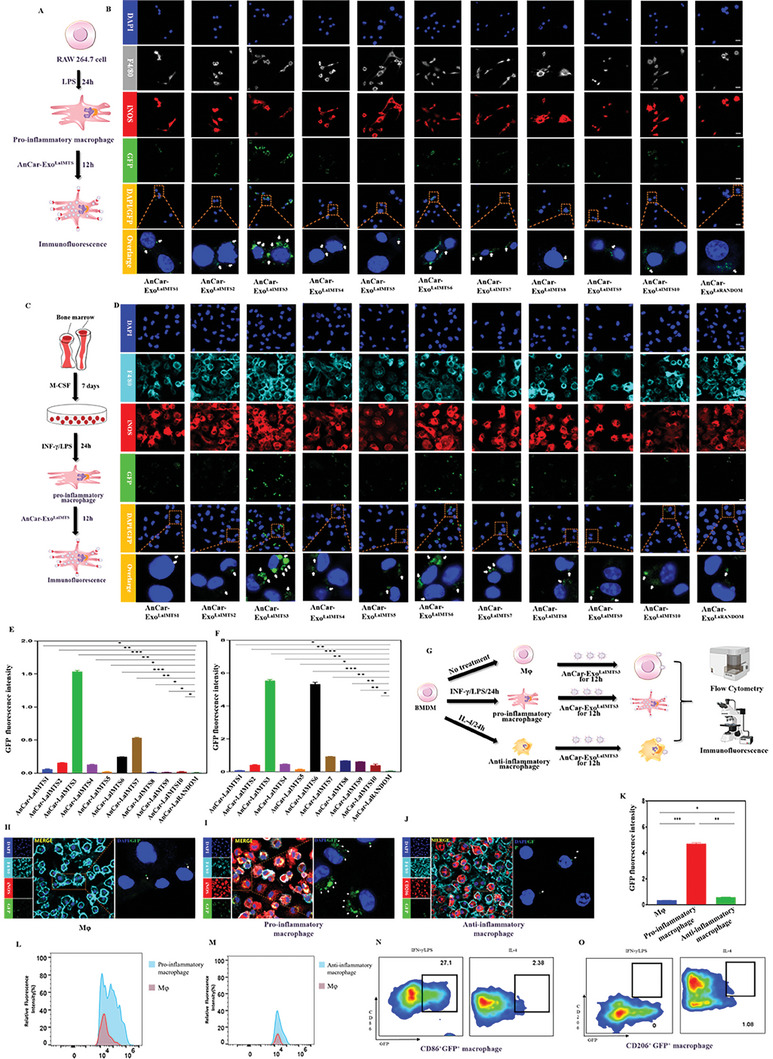
Screening and identification of the optimal AnCar‐Exo^LaIMTS^ that targets pro‐inflammatory macrophage in vitro. A) A schematic diagram illustrating that pro‐inflammatory macrophages derived from LPS‐activated RAW 264.7 cells were incubated with AnCar‐Exo^LaIMTS1^ to AnCar‐Exo^LaIMTS10^, AnCar‐Exo^LaRANDOM^ (1 × 10^8^ particles) for 12 h. B) The uptake of exosomes by pro‐inflammatory macrophages derived from RAW 264.7. Immunofluorescence showed F4/80 (gray), iNOS (red) and GFP (green) in exosome‐treated RAW 264.7 cells. Scale bars, 10 µm. C) A schematic diagram illustrating that how BMDM was separated from bone barrow and pro‐inflammatory macrophages derived from LPS/INF‐γ‐activated BMDM were incubated with AnCar‐Exo^LaIMTS1^ to AnCar‐Exo^LaIMTS10^, AnCar‐Exo^LaRANDOM^ (1 × 10^8^ particles) for 12 h. Scale bars, 10 µm. D) The uptake of exosomes by pro‐inflammatory macrophages induced from BMDM. Immunofluorescence showed F4/80 (sky blue), iNOS (red) and GFP (green) in exosome‐treated BMDM cells. Scale bars, 10 µm. E) Quantification of GFP fluorescence intensities in exosome‐treated RAW 264.7 cells. GFP fluorescence intensities were calculated under three high‐magnification fields with image J. N = 3 independent biological replicates, *P < 0.05, **P < 0.01 and ***P < 0.001. F) Quantification of GFP fluorescence intensities in exosome‐treated BMDM cells. GFP fluorescence intensities were calculated under three high‐magnification fields with image J. N = 3 independent biological replicates, *P < 0.05, **P < 0.01 and ***P < 0.001. G) A schematic diagram illustrating that how Mφ, pro‐inflammatory macrophage and anti‐inflammatory macrophage were induced from BMDM and detected by Immunofluorescence or Flow Cytometry after treated with AnCar‐Exo^LaIMTS3^. H, I and J) Immunofluorescence showed the uptake of AnCar‐Exo^LaIMTS3^ by Mφ, pro‐inflammatory macrophage and anti‐inflammatory macrophage. Scale bars, 10 µm. K) Quantification of GFP fluorescence intensities in AnCar‐Exo^LaIMTS3^treated Mφ, pro‐inflammatory macrophage and anti‐inflammatory macrophage. GFP fluorescence intensities were calculated under three high‐magnification fields with image J. N = 3 independent biological replicates, *P < 0.05, **P < 0.01 and ***P < 0.001. L, M) Flow Cytometry showed the ratio of relative fluorescence intensity between pro‐inflammatory macrophage and Mφ or between anti‐inflammatory macrophage and Mφ. N) Flow Cytometry shows the ratio of CD86^+^GFP^+^ in LPS/INF‐γ‐activated or IL‐4‐activated BMDM. O) Flow Cytometry shows the ratio of CD206^+^GFP^+^ in LPS/INF‐γ‐activated or IL‐4‐activated BMDM. All values were displayed as the way of mean ± SD. One‐way analysis of variance (ANOVA) was used to evaluate the difference among groups.

In order to screen and identify the optimal AnCar‐Exo^LaIMTS^ that targets pro‐inflammatory macrophages with high efficiency, confocal microscopy was used to observe the uptake of exosomes by pro‐inflammatory macrophages. RAW 264.7 cells were stimulated with 100 ng ml^−1^ LPS for 24 h to induce pro‐inflammatory transition. AnCar‐Exo^LaIMTS1^ to AnCar‐Exo^LaIMTS10^, AnCar‐Exo^LaRANDOM^ were then added into culture medium and co‐cultured with pro‐inflammatory macrophages for 12 h (**Figure** **4**A). As shown in Figure 4B, AnCar‐Exo^LaIMTS1^ to AnCar‐Exo^LaIMTS10^ as well as AnCar‐Exo^LaRANDOM^ could enter into pro‐inflammatory macrophages with variable targeting abilities. Interestingly, AnCar‐Exo^LaIMTS3^ had the best affinity toward pro‐inflammatory macrophages, whose fluorescence intensity was higher than that of other AnCar‐modified exosomes. Furthermore, 12 h after the incubation of those AnCar‐Exosomes (AnCar‐Exo^LaIMTS1^ to AnCar‐Exo^LaIMTS10^, AnCar‐Exo^LaRANDOM^) with pro‐inflammatory bone marrow‐derived macrophages (BMDM) induced by LPS and INF‐γ (Figure 4C,E), AnCar‐Exo^LaIMTS3^ showed the highest uptake by pro‐inflammatory BMDM (Figure 4D,F), whose fluorescence intensity was also higher than that of other AnCar‐modified exosomes. Above all, AnCar‐Exo^LaIMTS3^ was an optimal AnCar‐Exo^LaIMTS^ that can specifically target pro‐inflammatory macrophages in vitro compared with other AnCar‐Exo^LaIMTS^.

We further evaluated the targeting ability of AnCar‐Exo^LaIMTS3^ toward other subtypes of macrophages. BMDM was induced in different subtypes of macrophages. Each subtype was incubated with AnCar‐Exo^LaIMTS3^ for 12 h and then examined by confocal microscopy and flow cytometry (Figure 4G). AnCar‐Exo^LaIMTS3^ could enter into other subtypes of macrophages, but the fluorescence intensity in Mφ and anti‐inflammatory macrophages was mild and showed significant decreases (Figure 4H–M). In addition, the data from flow cytometry revealed that the percentage of CD86^+^GFP^+^ cells (representing pro‐inflammatory macrophages) ingesting AnCar‐Exo^LaIMTS3^ was 27.1% in LPS and INF‐γ stimulated BMDM, while it was 1.08% for CD206^+^GFP^+^ cells (represent anti‐inflammatory macrophages) in IL‐4 stimulated BMDM (Figure 4N–Q). These data demonstrated that AnCar‐Exo^LaIMTS3^ can be largely enriched in pro‐inflammatory macrophages compared with Mφ or anti‐inflammatory macrophages. In addition, the primary chondrocytes and synovial fibroblasts were also tested for the targeting ability of AnCar‐Exo^LaIMTS3^. Data of Figure S1, (Supporting Information) indicated that only very few chondrocytes and synovial fibroblasts showed green fluorescence signals after incubated with AnCar‐Exo^LaIMTS3^ for 12 h. In brief, AnCar‐Exo^LaIMTS3^ is the most optimal AnCar‐Exo^LaIMTS^ targeting pro‐inflammatory macrophages.

### 2.4 Evaluating the Distribution and Potential Toxicity of AnCar‐Exo^LaIMTS3^ In Vivo

**Figure 5:**
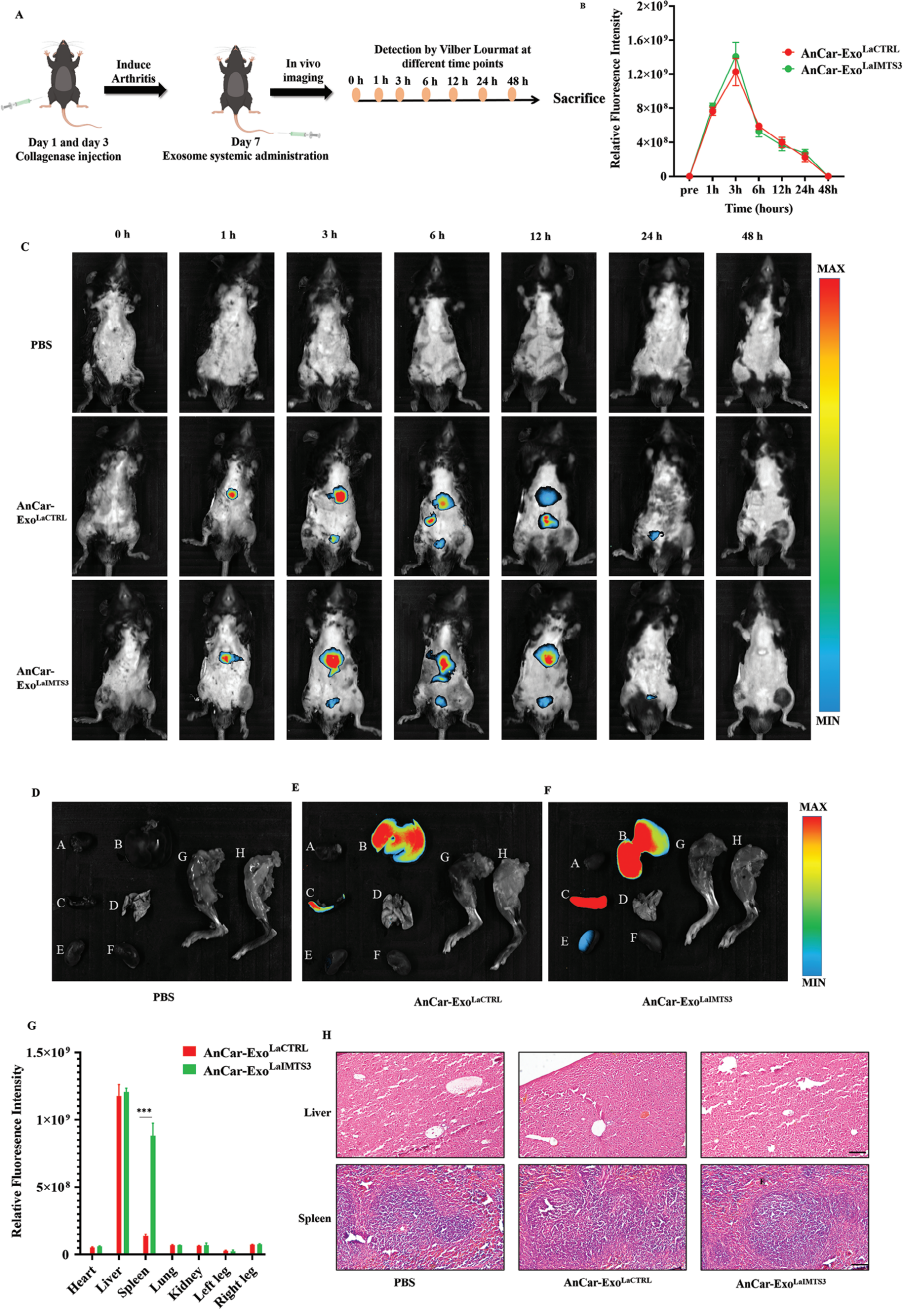
Evaluating the distribution and potential toxicity of AnCar‐Exo^LaIMTS3^ in vivo. A) A schematic diagram illustrating that the C57 mice were induced arthritis by collagenase and injected with AnCar‐Exo^LaIMTS3^ through systemic administration. B) Quantification of whole‐ body DID fluorescence intensities in mice treated with AnCar‐Exo^LaIMTS3^ and AnCar‐Exo^LaCTRL^. All values were displayed as the way of mean ± SD. Two‐way analysis of variance (ANOVA) was used to evaluate the difference among groups. N = 10 per group. C) Representative images of mice treated with PBS or exosomes and detected by Vilber Lourmat at different time points. D–F) DID fluorescence in different organs of mice at 3 h after exosome injection. A: Heart, B: Liver, C: Spleen, D: Lung, E: Left Kidney, F: Right Kidney, E: Left Leg, H: Right Leg. N = 3 per group. G) Quantification of DID fluorescence intensities in different organs between AnCar‐Exo^LaIMTS3^ and AnCar‐Exo^LaCTRL^ at 3 h after injection, N = 3 per group. All values were displayed as the way of mean ± SD. One‐way analysis of variance (ANOVA) was used to evaluate the difference among groups. ***P < 0.001. H) Hematoxylin‐eosin staining of liver and spleen at 48 h after injection. N = 3 per group. Scale bars, 100 µm.

Next, we evaluated the distribution and potential toxicity of AnCar‐Exo^LaIMTS3^ in vivo, which is important for its safe application in future. AnCar‐Exo^LaCTRL^ and AnCar‐Exo^LaIMTS3^ were labelled by DID dye and delivered into collagenase‐induced‐arthritis mice through systemic administration. Then the distribution of exosomes was detected by Vilber Lourmat at different time points (0, 1, 3, 6, 12, 24, and 48 h) (**Figure** **5**A). As shown in Figure 5B,C, DID fluorescence could be detected in mice injected with AnCar‐Exo^LaCTRL^ and AnCar‐Exo^LaIMTS3^, but not in those with PBS administration. Moreover, the DID fluorescence intensity in mice with AnCar‐Exo^LaCTRL^ or AnCar‐Exo^LaIMTS3^ gradually increased after administration and achieved the peak intensity at 3 h. It was subsequently decreased and almost disappeared at 48 h, suggesting that AnCar‐Exo^LaCTRL^ and AnCar‐Exo^LaIMTS3^ had similar metabolic rates in vivo. Furthermore, we explored the distribution of exosomes in different organs of mice at 3 h after systemic administration. As shown in Figure 5D–G, the fluorescence of AnCar‐Exo^LaCTRL^ and AnCar‐Exo^LaIMTS3^ was mainly detected in liver, spleen and kidney. Interestingly, the fluorescence intensities of AnCar‐Exo^LaIMTS3^ in spleen were stronger than that of AnCar‐Exo^LaCTRL^, while it didn't show significant differences between those two groups in liver and kidney. In addition, hematoxylin‐eosin staining revealed that AnCar‐Exo^LaIMTS3^ didn't result in pathological injuries such as cell death and fibrosis in heart, liver, spleen, lung and kidney, suggesting that AnCar‐Exo^LaIMTS3^ didn't induce remarkable toxicity in these organs (Figure 5H; Figure S2, Supporting Information). In brief, AnCar‐Exo^LaIMTS3^ is mainly distributed in liver, spleen and kidney with no significant toxicity in mice.

### 2.5 AnCar‐Exo^LaIMTS3^ Could Specifically Target Pro‐Inflammatory Macrophages in Arthritic Mice

**Figure 6:**
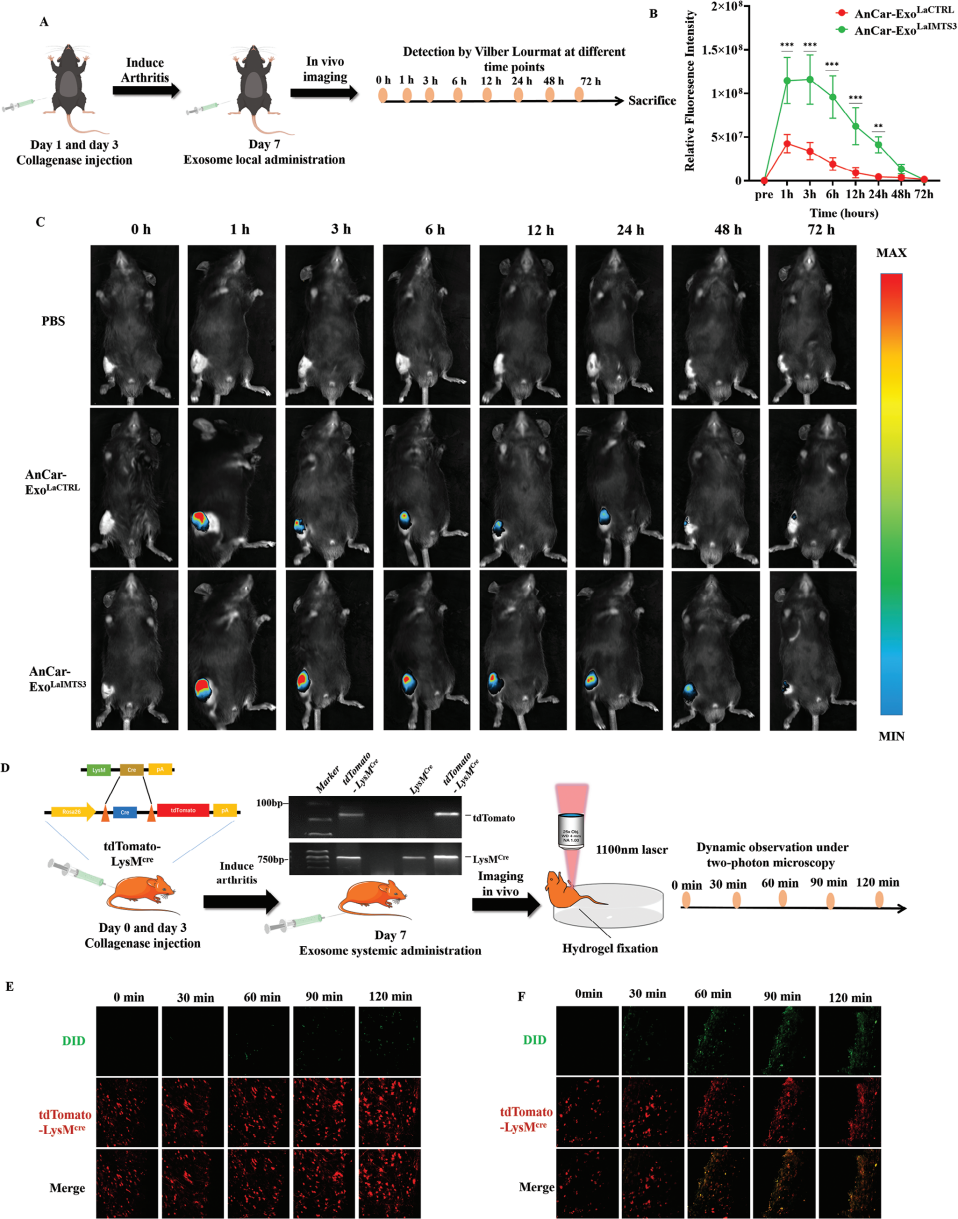
AnCar‐Exo^LaIMTS3^ could specifically target synovial macrophage in mice of inflammatory arthritis. A) A schematic diagram illustrating that the C57 mice were induced arthritis by collagenase and intra‐articularly injected with AnCar‐Exo^LaIMTS3^ and AnCar‐Exo^LaCTRL^ exosomes. B) Quantification of joint DID fluorescence intensities in mice treated with AnCar‐Exo^LaIMTS3^ and AnCar‐Exo^LaCTRL^. All values were displayed as the way of mean ± SD. Two‐way analysis of variance (ANOVA) was used to evaluate the difference among groups. N = 10 per group, **P < 0.01, ***P < 0.001. C) Representative images of mice treated with PBS or exosomes through intra‐articular injection and detected by Vilber Lourmat at different time points. D) A schematic diagram illustrating establishment of macrophage labelling *tdTomato‐LysM^Cre^* mice and time‐series in vivo imaging of exosomes entering into macrophages as detected by two‐photon microscopy. E, F) the uptake of DID labeled AnCar‐Exo^CTRL^ and AnCar‐Exo^LaIMTS3^ by tdTomato positive synovial macrophages.

There was a large amount of activated macrophages in synovial tissues of inflammatory arthritis, which greatly contributed to joint injury. Our previous studies indicate that targeting these synovial macrophages is a potent strategy for the treatment of arthritis.^[^ ^14^ ^]^ Then, we further evaluated whether AnCar‐Exo^LaIMTS3^ could specifically target synovial macrophages in inflammatory arthritis. First, we observed the distribution of intra‐articularly injected AnCar‐Exo^LaIMTS3^ by Vilber Lourmat at different time points following injection (0, 1, 3, 6, 12, 24, 48, and 72 h) (**Figure** **6**A). As shown in Figure 6B, AnCar‐Exo^LaIMTS3^, but not the AnCar‐Exo^LaCTRL^, was significantly enriched in inflammatory joints. Statistically, the fluorescence intensity in AnCar‐Exo^LaIMTS3^ treatment group was notably higher than that of AnCar‐Exo^LaCTRL^ (Figure 6C). In addition, the intensity of AnCar‐Exo^LaIMTS3^ reached the peak value at 1 h and almost disappeared at 72 h, while the intensity in AnCar‐Exo^LaCTRL^ nearly disappeared at 48 h. These results indicated that AnCar‐Exo^LaIMTS3^ could be enriched in inflammatory joints with higher intensity and longer retention time than that of AnCar‐Exo^LaCTRL^.

Next, we detected the targeting ability of AnCar‐Exo^LaIMTS3^ for synovial macrophages in arthritic mice by in vivo imaging. Briefly, *tdTomato‐ LysM^Cre^* mice were used to label synovial macrophages,^[^ ^15^ ^]^ which were further polarized into pro‐inflammatory macrophages after collagen‐induced arthritis, then the targeting ability of systematically administrated AnCar‐Exo^LaCTRL^ and AnCar‐Exo^LaIMTS3^toward pro‐inflammatory synovial macrophages were identified by two‐photon microscopy in vivo (Figure 6D). As shown in Figure 6E and Movie S1 (Supporting Information), some of the green fluorescence (AnCar‐Exo^LaCTRL^) gradually entered the synovial tissue of arthritic mice, hardly seen any exosomes co‐localized with synovial macrophages by the time 2 h after exosome administration. On the contrary, when arthritic mice were treated with AnCar‐Exo^LaIMTS3^ for 1 h, there were significantly more exosomes, residing on synovial tissue, which was largely co‐localized with macrophages (Figure 6F and Movie S1, Supporting Information). All the above data indicated that AnCar‐Exo^LaIMTS3^ could cause pro‐inflammatory macrophages in arthritic joints.

At last, we observed the co‐localization of AnCar‐Exo^LaIMTS3^ with pro‐inflammatory macrophages after their systemic administration in joint. As shown in Figure S3A,B (Supporting Information), after mice were intra‐articularly treated with AnCar‐Exo^LaIMTS3^ and AnCar‐Exo^LaCTRL^,the DID fluorescence of AnCar‐Exo^LaIMTS3^ group, but not AnCar‐Exo^LaCTRL^ group, was significantly increased in synovium and largely colocalized with F4/80^+^iNOS^+^ positive cells. The ratio of DID positive cells in F4/80+iNOS+ cells was significantly higher than that in F4/80^+^iNOS^−^ cells (none pro‐inflammatory macrophages) in AnCar‐Exo^LaIMTS3^ group, while it presented no difference in AnCar‐Exo^LaCTRL^ group (Figure S4A–D, Supporting Information). These results suggest that pro‐inflammatory macrophages are the major subtype of synovial macrophages in collagenase‐induced‐arthritis. While AnCar‐Exo^LaIMTS3^ could be largely enriched in these pro‐inflammatory macrophages. Collectively, the synovial pro‐inflammatory macrophages were the main targeted cells of AnCar‐Exo^LaIMTS3^ in the collagenase‐induced‐ arthritis.

### 2.6 AnCar‐Exo^LaIMTS3^ Could Specifically Target Pro‐Inflammatory Macrophage in Caudal fin Injury Model of Zebrafish

**Figure 7:**
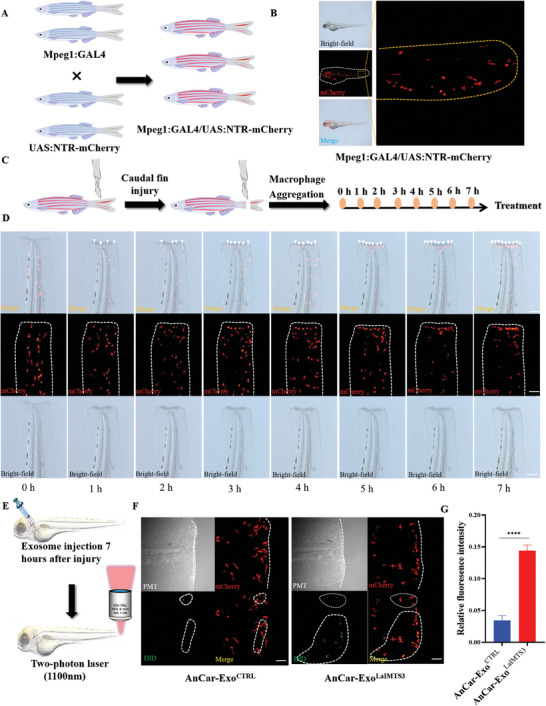
AnCar‐Exo^LaIMTS3^ could specifically target recruited macrophage in zebrafish caudal fin injury model. A) A schematic diagram showing the establishment of macrophage labeling transgenic zebrafish. B) Genotype identification using mCherry protein under fluorescence microscope. C) A schematic diagram showing the caudal fin injury model and macrophage aggregation during 7 h. D) Injury to the caudal fin leads to the aggregation of macrophages at the injured site. E) A schematic diagram illustrating the entry of exosome into macrophage detected by two‐photon microscopy. F, G) Two‐photon microscopy showed that mCherry‐labeled macrophages ingested exosome labelled by DID dye during 5 min. N = 3 independent biological replicates, Scale bars, 50 µm. ***P < 0.001. All values were displayed as the way of mean ± SD. One‐way analysis of variance (ANOVA) was used to evaluate the difference among groups.

Next, we test the targeting ability of AnCar‐Exo^LaIMTS3^ toward pro‐inflammatory macrophages that were recruited in the injury site of zebrafish caudal fin.^[^ ^16^ ^]^ First, Mpeg1:GAL4 and UAS: NTR‐mCherry lineages were crossed to obtain the Mpeg1:GAL4/UAS:NTR‐mCherry transgenic zebrafishes(**Figure** **7**A), which often were used to trace macrophage.^[^ ^16c^ ^]^ Through fluorescence tracking in vivo, we observed macrophages with red fluorescent signals in those transgenic zebrafishes (Figure 7B). Subsequently, the recruitment of pro‐inflammatory macrophages was detected by two‐photon microscopy at early stage after injury of caudal fin in transgenic zebrafish (Figure 7C). As shown in Figure 7D, the red fluorescence labeled macrophages were gradually recruited into the injured site during 7 h after injury of caudal fin, which indicated that a lot of pro‐inflammatory macrophages were accumulated at early stage after injury. Then, we observed the green fluorescence of AnCar‐Exo^LaIMTS3^ in the above pro‐inflammatory macrophages through intracardiac injection (Figure 7E). As shown in Figure S5 (Supporting Information), AnCar‐Exo^LaIMTS3^ could enter into blood circulation system and reached the injured site of caudal fin in zebrafish. Moreover, the representative images in Figure 7F showed that the green fluorescence of AnCar‐Exo^LaIMTS3^ had more co‐localization with red fluorescence compared to AnCar‐Exo^CTRL^. The statistical analysis also showed similar results (Figure 7G), suggesting that AnCar‐Exo^LaIMTS3^ showed higher targeting ability toward macrophages. To sum up, our data demonstrate that the AnCar‐Exo^LaIMTS3^ could specifically target pro‐inflammatory macrophages in caudal fin injury model of zebrafish.

### 2.7 Identify the Cellular Uptake Mechanisms of AnCar‐Exo^LaIMTS3^ by Pro‐Inflammatory Macrophage

**Figure 8:**
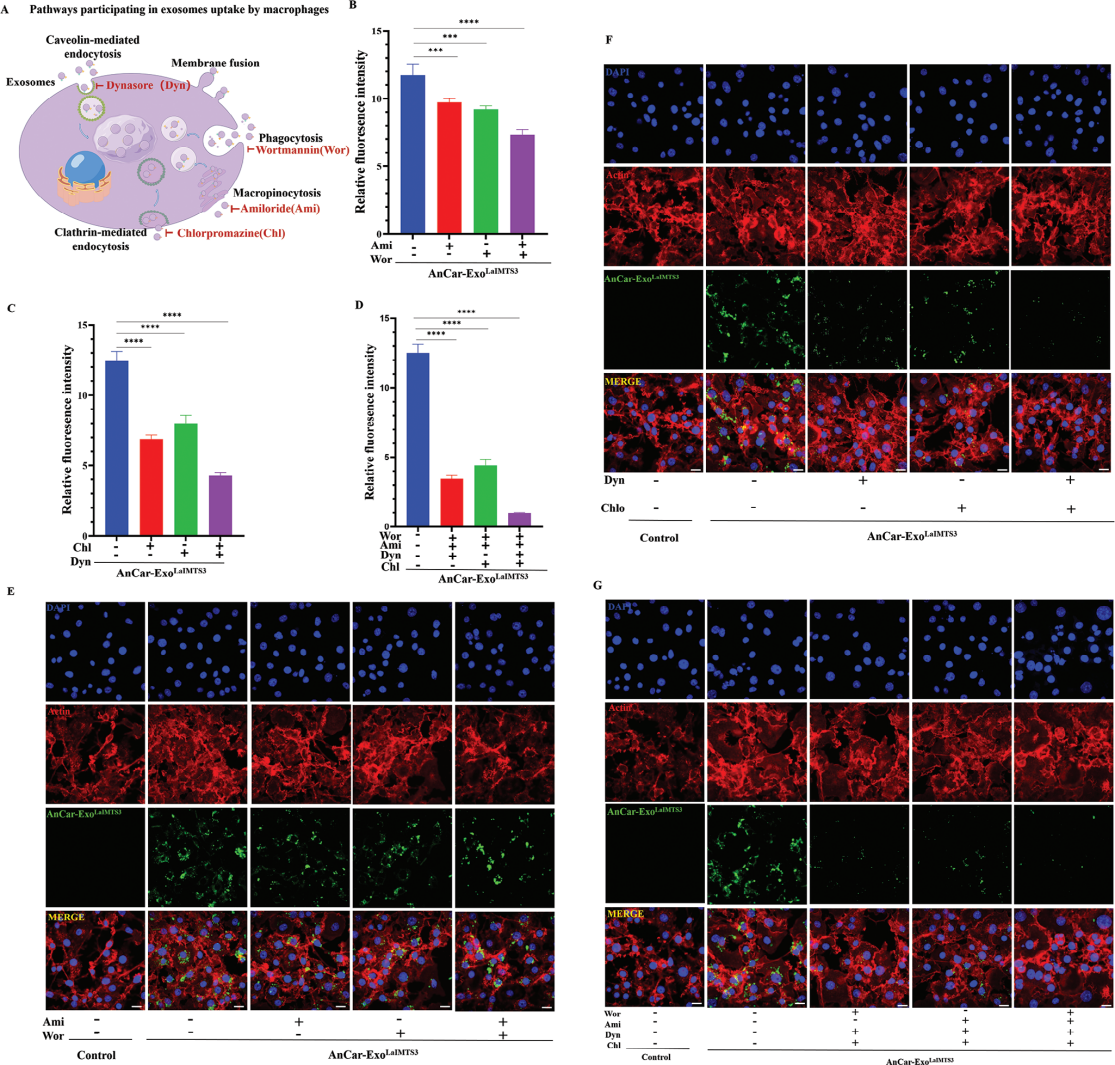
Identify the cellular uptake mechanisms of AnCar‐Exo^LaIMTS3^ targeting pro‐inflammatory macrophage. A) Potential patterns of pro‐inflammatory macrophage uptaking exosomes. B) Quantification of AnCar‐Exo^LaIMTS3^ fluorescence intensities in pro‐inflammatory macrophage after phagocytosis (inhibitor: Wortmannin, Wor) and macropinocytosis (inhibitor: Amiloride, Ami) were inhibited. The fluorescence intensities were calculated under three high‐magnification fields with image J. N = 3 independent biological replicates, ***P < 0.001, ****P < 0.0001. C) Quantification of AnCar‐Exo^LaIMTS3^ fluorescence intensities in pro‐inflammatory macrophage after the caveolin‐mediated endocytosis (inhibitor: Dynasore, Dyn) and clathrin‐mediated endocytosis (inhibitor: Chlorpromazine, Chl) were inhibited. The fluorescence intensities were calculated under three high‐magnification fields with image J. N = 3 independent biological replicates, ****P < 0.0001. D) Quantification of AnCar‐Exo^LaIMTS3^ fluorescence intensities in pro‐inflammatory macrophage after phagocytosis, macropinocytosis, caveolin‐mediated endocytosis and clathrin‐mediated endocytosis were inhibited. The fluorescence intensities were calculated under three high‐magnification fields with image J. N = 3 independent biological replicates, ****P < 0.0001. E) Representative images of pro‐inflammatory macrophages uptake AnCar‐Exo^LaIMTS3^ when phagocytosis and macropinocytosis were inhibited. Scale bars, 20 µm. F) Representative images of pro‐inflammatory macrophages uptake AnCar‐Exo^LaIMTS3^ when the caveolin‐mediated endocytosis and clathrin‐mediated endocytosis were inhibited. Scale bars, 20 µm. G) Representative images of pro‐inflammatory macrophages uptake AnCar‐Exo^LaIMTS3^ when phagocytosis, macropinocytosis, caveolin‐mediated endocytosis and clathrin‐mediated endocytosis were inhibited. Scale bars, 20 µm. All values were displayed as the way of mean ± SD. One‐way analysis of variance (ANOVA) was used to evaluate the difference among groups.

Numerous pathways participate in cellular uptake of exosomes by cells, including phagocytosis, membrane fusion, macropinocytosis, caveolin‐mediated endocytosis, and clathrin‐mediated endocytosis^[^ ^17^ ^]^ (**Figure** **8**A). To further investigate the cellular uptake mechanisms, we used small‐molecule inhibitors of those aforementioned pathways in subsequent experiments. As shown in Figure 8B, the green fluorescence (AnCar‐Exo^LaIMTS3^) in pro‐inflammatory macrophages slightly attenuated with phagocytosis or macropinocytosis inhibitor. This data revealed that the phagocytosis and macropinocytosis partially participated in cellular uptake of AnCar‐Exo^LaIMTS3^ by pro‐inflammatory macrophages. Additionally, the green fluorescence in pro‐inflammatory macrophages was notably attenuated with caveolin or clathrin inhibitors. Interestingly, the reduction of AnCar‐Exo^LaIMTS3^ enrichment caused by caveolin or clathrin inhibition surpassed that from phagocytosis and macropinocytosis inhibition (Figure 8C). Furthermore, we inhibited caveolin or clathrin‐mediated endocytosis while phagocytosis or macropinocytosis was inhibited. The results displayed a significant reduction in green fluorescence enrichment within pro‐inflammatory macrophages, especially when both caveolin and clathrin were inhibited. This showed the crucial role of caveolin or clathrin‐mediated endocytosis in cellular uptake mechanism of AnCar‐^ExoLaIMTS3^ toward pro‐inflammatory macrophages (Figure 8D). The representative figures was shown in Figure 8E,F. Similarly, Previous studies have reported that Caveolin and clathrin‐mediated endocytosis can dramatically enhance the endocytosis efficacy of specific macromolecules, often surpassing phagocytosis and macropinocytosis rates by more than 1000‐fold.^[^ ^18^ ^]^ Therefore, the targeting ability of AnCar‐Exo^LaIMTS3^ toward pro‐inflammatory macrophage was mainly achieved through the caveolin and clathrin‐mediated endocytosis.

### 2.8 AnCar‐Exo^LaIMTS3^ Targets Pro‐Inflammatory Macrophage Mainly Through Toll‐like Receptor 4 (TLR4)

**Figure 9:**
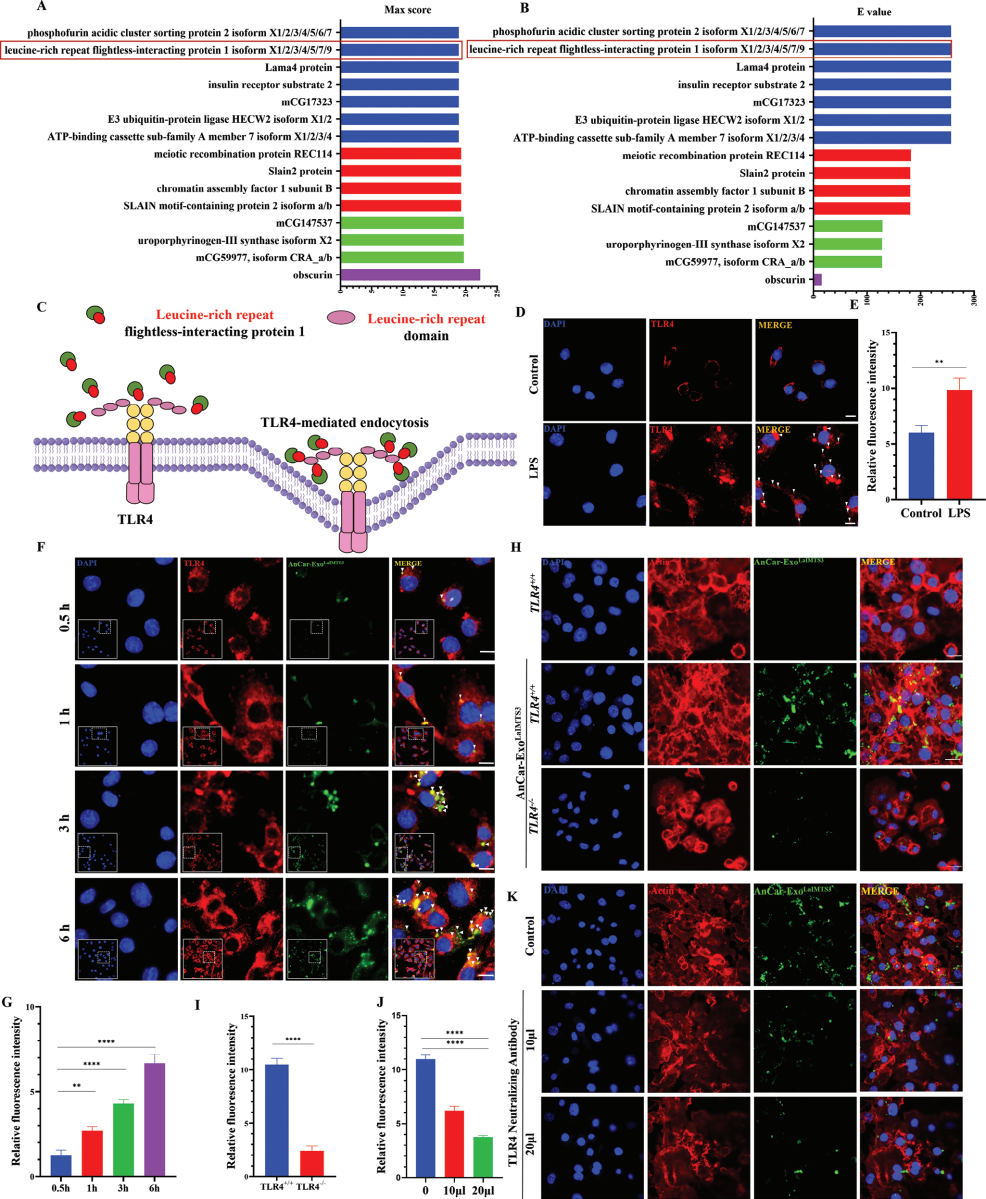
AnCar‐Exo^LaIMTS3^ targeted pro‐inflammatory macrophage through Toll‐like receptor 4 (TLR4). A) The Max score of protein blast in NCBI. B) The E value of protein blast in NCBI. C) A schematic diagram that Leucine‐rich repeat flightless‐interacting protein 1 binds to Leucine‐rich repeat domain. D) Immunofluorescence showed the number of TLR4 activated by LPS increased. Scale bars, 20 µm. E) Quantification of TLR4 fluorescence intensities in macrophages between control and LPS group. The fluorescence intensities were calculated under three high‐magnification fields with image J. N = 3 independent biological replicates, **P < 0.01. F) Immunofluorescence showed the co‐localization of AnCar‐Exo^LaIMTS3^ and TLR4 gradually increased within 6 hours. Scale bars, 20 µm. G) Quantification of the co‐localization fluorescence intensities between AnCar‐Exo^LaIMTS3^ and TLR4 in macrophages between control and LPS group. The fluorescence intensities were calculated under three high‐magnification fields with image J. N = 3 independent biological replicates, **P < 0.01, ****P < 0.0001. H) Immunofluorescence showed AnCar‐Exo^LaIMTS3^ in TLR4^−/−^ and TLR4^+/+^ BMDM. Scale bars, 20 µm. I) Quantification of AnCar‐Exo^LaIMTS3^ fluorescence intensities in TLR4^−/−^ and TLR4^+/+^ BMDM. The fluorescence intensities were calculated under three high‐magnification fields with image J. N = 3 independent biological replicates, ****P < 0.0001. J) Quantification of AnCar‐Exo^LaIMTS3^ fluorescence intensities in macrophages treated by TLR4 neutralizing antibody. The fluorescence intensities were calculated under three high‐magnification fields with image J. N = 3 independent biological replicates, ****P < 0.0001. K) Immunofluorescence showed the enrichment level of exosomes in peritoneal pro‐inflammatory macrophages decreases as the concentration of TLR4‐neutralizing antibodies increases, Scale bars, 20 µm. All values were displayed as the way of mean ± SD. One‐way analysis of variance (ANOVA) was used to evaluate the difference among groups.

It was reported that caveolin‐mediated and clathrin‐mediated endocytosis were closely associated with receptor‐mediated uptake processes.^[^ ^19^ ^]^ Moreover, receptor‐mediated endocytosis can enhance the selective uptake of exosomes by cells.^[^ ^18d^ ^]^ Therefore, we deduced that receptor‐mediated endocytosis may play an important role in targeting ability of AnCar‐Exo^LaIMTS3^ toward pro‐inflammatory macrophages. Next, we investigated the potential key receptor which was related to receptor‐mediated endocytosis of AnCar‐Exo^LaIMTS3^ by pro‐inflammatory macrophage. First, we conducted a comprehensive screen of protein data sourced from the NCBI protein database, specifically focusing on proteins harboring the amino acid sequence aligned with AnCar‐LaIMTS3. As shown in Supplementary Materials Excel 1, certain proteins were identified containing the sequence of LPSSGAA, based on the Max score and E value criteria. Among these candidates, we further focused on the leucine‐rich repeat flightless‐interacting protein 1(**Figure** **9**A,B), which was a positive regulator of TLR4 in innate inflammatory responses.^[^ ^20^ ^]^ Interestingly, the extracellular fragments of TLR4 consisted of leucine‐rich repeats domain, which could interact with leucine‐rich repeat flightless‐interacting protein 1^[^ ^20^, ^21^ ^]^(Figure 9C). A previous study reported an increase in the expression of TLR4 pro‐inflammatory macrophage.^[^ ^22^ ^]^ Similarly, our data also showed that the red fluorescence‐labeled with TLR4 antigen in LPS‐treated macrophages exhibited a significant up‐regulation compared to the control group, suggesting an increased expression of TLR4 protein in pro‐inflammatory macrophages (Figure 9D,E). Then, we observed the colocalization of TLR4 and AnCar‐Exo^LaIMTS3^ in pro‐inflammatory macrophages at different time points (0.5, 1, 3, and 6 h) using immunofluorescence assay. As shown in Figure 9F,G, the intensity of co‐localized fluorescence (yellow) was gradually increased with incubation time, indicating that AnCar‐Exo^LaIMTS3^ may enter into pro‐inflammatory macrophages through TLR4‐related endocytosis pathway. To further clarify the role of TLR4 in cellular uptake of AnCar‐Exo^LaIMTS3^, we isolated peritoneal macrophages (PM) from TLR4^−/−^ mice, and detected the change of AnCar‐Exo^LaIMTS3^ fluorescence signals in TLR4^−/‐^ PM treated with LPS and IFN‐γ. As indicated in Figure 9H, the size of nucleus in TLR4^−/‐^ PM under LPS/IFN‐γ stimuli was smaller than the ones from wild‐type mice, suggesting TLR4 was essential for activation of macrophages in this condition. Moreover, the green fluorescence signal of AnCar‐Exo^LaIMTS3^ can hardly be observed in LPS/IFN‐γ‐treated TLR4^−/‐^ PM. The statistical analysis also revealed that green fluorescence intensity of AnCar‐Exo^LaIMTS3^ was obviously decreased in TLR4^−/‐^ PM compared with control ones (Figure 9I). Moreover, we added TLR4‐neutralizing antibody in the cell culture medium to antagonize the TLR4‐mediated endocytosis pathway. As shown in Figure 9J,K, the enrichment of green fluorescence within pro‐inflammatory macrophages was significantly decreased after the treatment of TLR4‐neutralizing antibody, further revealing that the targeting ability of AnCar‐Exo^LaIMTS3^ toward pro‐inflammatory macrophages was mainly mediated by the TLR4. In conclusion, TLR4‐mediated endocytosis plays a key role in the targeting ability of AnCar‐Exo^LaIMTS3^ toward pro‐inflammatory macrophages.

### 2.9 Targeting HIF‐1α in Synovial Macrophages by AnCar‐Exo^LaIMTS3^ Ameliorated the Severity of Inflammatory Arthritis

**Figure 10:**
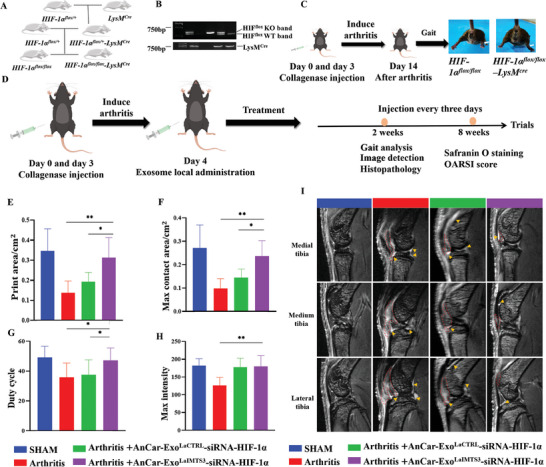
Targeting HIF‐1α in synovial macrophage by AnCar‐Exo^LaIMTS3^ ameliorated the severity of inflammatory arthritis. A) A schematic diagram illustrating the establishment of transgenic mouse model with HIF‐1α conditional deletion in the macrophages. B) Genotype identification using PCR. C) The strategy of inducing arthritis by injection collagenase and phenotype between HIF‐1α^flox/flox^ and HIF‐1α^flox/flox^‐LysM^cre^ transgenic mice. n = 5 per group. D) A schematic diagram illustrating targeting HIF‐1α in synovial macrophage by AnCar‐Exo^LaIMTS3^ to treatment arthritis and detection by gait analysis, medical image and histopathology. E–H) The changes of gait parameters among groups (n = 5 per group), including Print area, Max contact area, Duty cycle, Max intensity. The values of the right hind parameters were displayed, N = 5 per group, *P < 0.05, **P < 0.01. I) Medial, medium and lateral tibial MRI images of right legs among groups. The red dotted line marks the articular capsule tissue. J, K) Synovitis was shown by H&E staining and Synovitis scores after 2 weeks’ treatment among groups. N = 5 per group. *P < 0.05. L) Immunofluorescence of F4/80 (green), HIF‐1α (red) in synovial tissues after treatment. Scale bars, 10 µm. M) Quantification analysis of F4/80 positive cells among groups. The fluorescence intensities were calculated under three high‐magnification fields with image J, N = 3 independent biological replicates, *P < 0.05, ***P < 0.001. N) Quantification analysis of F4/80^+^HIF‐1α^+^ cells among groups. The fluorescence intensities were calculated under three high‐magnification fields with image J, N = 3 independent biological replicates, *P < 0.05, ***P < 0.001. O–Q) Representative images of Safranin O/Fast Green–stained sections of knee joints among groups after 8 weeks’ treatment. N = 5 per group. Scale bar, 100 µm. The severity of articular cartilage damage among groups were evaluated using the OARSI scoring system, maximal and summed scores were calculated, *P < 0.05, ***P < 0.001. All values were displayed as the way of mean ± SD. One‐way analysis of variance (ANOVA) was used to evaluate the difference among groups.

Previous studies have reported that HIF‐1α plays an important role in macrophage activation and deletion of HIF‐1α in macrophage could dramatically alleviate inflammatory response in arthritis induced by collagen.^[^ ^23^ ^]^ Here, we crossed HIF‐1α^flox/+^ mice with the LysM^cre^ transgenic mice to generate mice (HIF‐1α^flox/flox^‐LysM^cre^) with HIF‐1α deletion in the macrophages as well as the corresponding controls (HIF‐1α^flox/flox^) (**Figure** **10**A,B). Subsequently, these mice were further used for arthritis induction and evaluation. As shown in Figure 8C, the arthritic joint of HIF‐1α^flox/flox^ mice displayed moderate damage after intra‐articular injection of collagenase for 14 days, which was attenuated in HIF‐1α^flox/flox^‐LysM^cre^ mice (Movie S2, Supporting Information). In addition, the synovium of HIF‐1α^flox/flox^ mice was thickened with higher synovitis score compared with that of HIF‐1α^flox/flox^‐LysM^cre^ mice (Figure S6A,B, Supporting Information), suggesting that HIF‐1α of macrophages is a key molecular target for the treatment of inflammatory arthritis. Therefore, we intended to specifically downregulate HIF‐1α of pro‐inflammatory synovial macrophages through AnCar‐Exo^LaIMTS3^ mediated HIF‐1α siRNA delivery, with the purpose of reducing synovial inflammatory response of arthritic mice. We screened three sequences of siRNA‐HIF‐1α and evaluated the interference efficiency of siRNA‐HIF‐1α in LPS‐activated RAW 264.7 or LPS/IFN‐γactivated BMDM. The optimal siRNA HIF‐1α‐3 was chosen for the subsequent experiments (Figure S7A,B, Supporting Information). Also, we also conducted additional experiments to investigate the kinetics of knock‐down rate of the HIF‐1α‐siRNA loaded AnCar‐Exo^LaIMTS3^. Our data showed that treatment of pro‐inflammatory cells (264.7 RAW cells activated by LPS) with HIF‐1α‐siRNA loaded AnCar‐Exo^LaIMS3^ significantly reduced the level of HIF‐1α at 24, 48 and 96 h (Figure S7C, Supporting Information). According to previous studies^[^ ^11^, ^24^ ^]^ and our kinetics data, the AnCar‐Exo^LaIMTS3^ loaded with siRNA‐HIF‐1α (AnCar‐Exo^LaIMTS3^‐siRNA‐HIF‐1α) was injected into knee joint of arthritic mice once every three days, and then gait characterization, imaging and histopathological analysis were used to comprehensively evaluate the treatment efficiency of AnCar‐Exo^LaIMTS3^‐siRNA‐HIF‐1α on collagenase induced‐arthritis (Figure 10D). As shown in Figure 10E–H, the Print area, Max contact area, Duty cycle, Swing speed and Max intensity were increased, while Swing was reduced in the group of AnCar‐Exo^LaIMTS3^‐siRNA‐HIF‐1α compared with PBS treated group (Figure S8A,B, Supporting Information). Besides, the degree of changes in gait‐related parameters was higher after treatment by AnCar‐Exo^LaIMTS3^‐siRNA‐ HIF‐1α compared with AnCar‐Exo^LaCTRL^‐ siRNA‐HIF‐1α. In addition, MRI imaging showed that synovial fluid was increased accompanied by a thickened synovial membrane and increased area of subpatellar fat pad in PBS treated group compared with the Sham group. These changes were dramatically inhibited by the treatment of AnCar‐Exo^LaIMTS3^‐siRNA‐HIF‐1α, while the inhibitory effect was weaker in AnCar‐Exo^LaCTRL^‐siRNA‐HIF‐1α group (Figure 10I; Figure S8C, Supporting Information). Similarly, HE staining also suggests that the increased synovitis score in arthritic mice was more significantly reversed by AnCar‐Exo^LaIMTS3^‐siRNA‐HIF‐1α than that of AnCar‐Exo^LaCTRL^‐siRNA‐HIF‐1α (Figure 10J,K). Moreover, immunofluorescence staining revealed that the number of F4/80+ cells, as well as percentage of F4/80^+^HIF‐1α^+^cells, were more significantly decreased in inflammatory joint injected with AnCar‐Exo^LaIMTS3^‐siRNA‐HIF‐1α compared to that of AnCar‐Exo^LaCTRL^‐siRNA‐HIF‐1α treated group(Figure 10L–N), indicating the efficient konckdown of HIF‐1α in macrophage of inflammatory joint and reduction of infiltration of inflammatory cells in synovium. However, there were no significant changes including osteophyte formation and joint space narrowing among the four groups as revealed by X‐ray images at an early stage of arthritis (Figure S9, Supporting Information). In addition, the Safranin O/Fast Green staining exhibited that AnCar‐Exo^LaIMTS3^‐siRNA‐HIF‐1α didn't influence obviously cartilage destruction at two weeks after collagenase induced‐arthritis compared with the control (Figure S10, Supporting Information). These data, together with MRI results, indicated that the inflammatory response of the joint occurred earlier than the changes of bone structure and damage of cartilage at the early stage of collagenase‐induced‐arthritis, and the regulatory effect of AnCar‐Exo^LaIMTS3^‐siRNA‐HIF‐1α might be related to inhibition of joint inflammatory response. Moreover, we investigated the joint lesion at the late stage (8 weeks) of collagenase‐induced‐arthritis using the Safranin O/Fast Green staining. The data showed that the joint cartilage showed significant deterioration with the highest OARSI score in the arthritis group, which could be partially reversed by the treatment of AnCar‐Exo^LaIMTS3^‐siRNA‐HIF‐1α (Figure 10O–Q). In brief, targeted delivery of siRNA‐HIF‐1α to inflammatory macrophages by AnCar‐Exo^LaIMTS3^ can efficiently inhibit the expression of HIF‐1α in synovial macrophages, decrease synovial inflammatory response, reduce the lesion of cartilage and ameliorate gait abnormalities in mice arthritis model.

## 3 Discussion

Macrophages are heterogenic phagocytic cells that play distinct roles in multiple physiological and pathological processes.^[^ ^25^ ^]^ Targeting different types of macrophages has shown potent therapeutic effects in diseases, which has attracted great interest in recent years.^[^ ^26^ ^]^ TAMs (tumor‐associated macrophages) are responsible for the cancer microenvironment, influencing growth and progression of tumors.^[^ ^27^ ^]^ Weissleder et al. reported that β‐cyclodextrin nanoparticles controlled tumor growth and prevented tumor relapse by targeting TAMs.^[^ ^28^ ^]^ Besides, exosomes modified by IL‐4 receptor reprogrammed TAMs into pro‐inflammatory macrophage and inhibited tumor growth.^[^ ^10^ ^]^ Although many approaches have been developed to target alternatively activated macrophages (M2 macrophages), there are few researches on targeting pro‐inflammatory macrophages partially because of the non‐specificity phagocytosis.^[^ ^29^ ^]^ Interestingly, our data indicated that the phagocytosis efficiencies of pro‐inflammatory macrophages showed obvious differences among the exosomes modified by different types of AnCar‐LaIMTS. In the existing candidate range, the AnCar‐Exo^LaIMTS3^ has the best targeting ability to pro‐inflammatory macrophages in vitro and in vivo, indicating that modification of exosomes through biomolecular engineering is a prospective method to target pro‐inflammatory macrophages.

Except for targeting ability, efficient loading of cargo into exosomes is also a crucial way to improve their therapeutic effectiveness for diseases. Recently, researchers have shown an increased interest in cargo loading of exosomes by itself. Ryosuke et al engineered surface of exosome membrane by conjugating L7Ae to the C‐terminus of CD63 in order to make exosome itself carry more mRNA after the 3′‐untranslated region of mRNA is inserted into a C/D_box_ sequence.^[^ ^30^ ^]^ Thus, it is expected that modification of the C‐terminus of AnCar‐LaIMTS according to the respective treatment needed will make exosomes selectively load specific cargos and improve their therapeutic effects. In addition, it is available to improve inflammatory responsiveness of exosome release in arthritis. Park et al reported that exosomes modified by a thioketal linker‐embedded poly (ethylene glycol) enhanced the cellular uptake of ROS‐responsive exosomes after the production of excessive ROS in arthritis.^[^ ^31^ ^]^ Therefore, combined application of exosomes and biomaterials may further improve the therapeutic effects of exosomes.

Engineered exosomes can enhance targeting efficiency to specific cells by binding to ligand receptors, which could maximize effectiveness of drug delivery.^[^ ^32^ ^]^ There are various pathways for extracellular macromolecules to enter cells, among which receptor‐mediated endocytosis enables selective and efficient uptake of large molecules in engineered exosomes.^[^ ^17^, ^19^ ^]^ Previous study has reported that exosomes with membrane surface modified by PD‐L1 could target cells with high expression of PD‐1 receptor, which have therapeutic efficiency in both UC and psoriasis disease.^[^ ^33^ ^]^ In addition, exosomes engineered by high binding affinity could deliver drugs to HER2 receptor‐expressing tumors in vivo.^[^ ^34^ ^]^ Our data indicate that the expression of TLR4 receptor is increased in pro‐inflammatory macrophages which greatly contributed to the targeting ability of AnCar‐Exo^LaIMTS3^ towards pro‐inflammatory macrophages.

In conclusion, we construct new engineered exosomes that can efficiently target pro‐inflammatory macrophages to exert the effect of targeted treatment of arthritis in this study. Wherein, the optimal AnCar‐Exo^LaIMTS3^ can efficiently deliver therapeutic contents to pro‐inflammatory macrophages mainly through TLR4‐mediated endocytosis, which ameliorates the severity of arthritis without obvious toxicity. This work provides a novel perspective and potential strategy for targeting treatments of arthritis and other inflammatory diseases in the future.

## 4 Experimental Section

### Experimental Reagents

Dulbecco's Modified Eagle Medium (DMEM, high glucose, 11965092), DMEM/F12 and Fetal bovine serum (FBS, 10100147) was obtained from Gibco (USA). DPBS, 1% penicillin/streptomycin solution and 0.25% trypsin‐EDTA were obtained from HyClone (USA). Exosome‐depleted FBS was obtained from VivaCell (Israel, C3801‐0100). Si‐RNA transfection reagent was Advanced Transfection Regeant (Zeta Life, USA). The reagent transferring Si‐RNA into exosomes was Exo‐Fect Exosome Transfection Reagent (SBI, EXFT20A‐1). DID was purchased from beyotime (C1995S). The following antibodies were used in this study: mouse anti LAMP2 antibody (1:1000 for WB; Proteintech, 66301‐1‐Ig), rabbit anti‐TSG101, (1:1000 for WB; Proteintech, 28283‐1‐AP), rabbit anti‐CD63 (1:1000 for WB; Proteintech, 25682‐1‐AP), rabbit anti‐CD81 (1:1000 for WB; Proteintech, 66866‐1‐Ig), mouse anti‐β‐actin (1:1000 for WB; Proteintech, 66009‐1‐Ig), rat anti‐F4/80 antibody (1:100 for IF; Abcam, ab6640), rabbit anti‐iNOS (1:100 for immunofluorescence; Proteintech,18985‐1‐AP), rabbit anti‐HIF‐1α (1:100 for IHC‐P; Abcam, ab216842).

### Transgenic Mice and Genotyping

The Lysozyme‐Cre (*LysM^Cre^*) was a transgenic mouse strain used to label macrophages.^[^ ^14^, ^35^ ^]^ Rosa26‐tdTomato (*tdTomato*) mice express red fluorescent protein in Cre‐expressing cells as reported in the previous study.^[^ ^36^ ^]^ HIF‐1α conditional knockout (HIF‐1α*^flox/flox^*) mice were used in the previous research.^[^ ^37^ ^]^ The Toll‐like receptor 4 knockout (*TLR4^−/−^*) mice were obtained from the Jackson Laboratory (**Stock No**: 007227). To trace macrophages in vivo, *LysM^Cre^* mice were mated with *tdTomato* mice to obtain *tdTomato‐ LysM^Cre^* mice, which were used to observe the in vivo dynamic process of engineered exosomes targeting macrophages under two‐photon microscopy (Germany, ZEISS). To obtain mice with macrophage‐specific HIF‐1α deletion, *HIF‐1α^flox/flox^* mice were bred with *LysM^Cre^* mice to obtain *HIF‐1α^flox/flox^*‐*LysM^Cre^* mice (referenced as *HIF‐1α* cKO mice). Littermates carrying *HIF‐1α^flox/flox^* without Cre recombinase were regarded as Control mice. The genotyping was performed as previously described.^[^ ^35^ ^]^ The following primers were used for genotyping. The *LysM^Cre^* primer: 5′‐CCC AGAAATGCCAGA TTACG ‐3′, complementary sequence, 5′‐CTTGGGCTGCCAGAATTTCTC‐3′. The *HIF‐1α^flox/flox^* primer: 5′‐TGATGTGGGTGCTGGTGTC‐3′, complementary sequence, 5′‐TTGTGTTGGGGCAGTACT G‐3′. The *TLR4^−/−^* primer: 5′‐GCAAGTTTC TATATGCATTCTC‐3′, complementary sequence, CCTCCATTTCCAATAGGTAG. All animals were fed with a standard diet and maintained in standard housing conditions (pathogen‐free cages at constant temperature and humidity). The circadian time was ≈12 h. Male mice were used in control group and experiment groups with collagenase‐induced‐arthritis. All animal‐related experiments were approved by the Medical Ethics Committee of Army Medical University (AMUWEC20201030) and given permission by the Institutional Animal Care and Use Committee of Daping Hospital in Chongqing.

### Plasmid Construction

As previous study reported,^[^ ^13^ ^]^ three repeated pro‐inflammatory macrophages were designed targeting sequences (1–10, Table S1, Supporting Information) or random sequences in the extracellular domain between signal peptide and mature peptide of human LAMP2B(NP_054701.1) through a flexible peptide linker (GGGGS) and added GFP tag to the carboxy‐terminal region of LAMP2B recombinant protein. The sequences coding recombinant protein were fused into PLVX‐puro at the XhoI and BamHI sites. Lentivirus was produced by transfecting HEK293T cells transiently. AnCar sequences were synthesized, and inserted into PLVX‐puro vector with XhoI and BamHI restriction sites. For the lentivirus package, HEK‐293T cells were transfected with the above plasmids, the psAX2 packaging plasmid and pMD2G envelope plasmid for 48 h to obtain the lentivirus supernatant.

### Establishment of Stable Cell Lines

HEK293T cells were cultured in a 6‐well plate at the density of 30–50% at day 1. Next, those Lentiviruses with anti‐puromycin gene were added according to the MOI of HEK293T (MOI = 5) in the afternoon on day 2. After the density of cells reached over 80%, the complete medium was replaced with fresh complete medium containing 2 µg ml^−1^ puromycin. When cell death didn't occur in six‐in well plate on day 5, the concentration of puromycin was reduced to 0.5 µg ml^−1^. Then, the mixed clonal cell lines were digested with 0.05% trypsin and counted. 60 cells cultured in 10 ml complete media were seeded in a 96‐well plate. After 10 days, the stable monoclonal cell line was passaged and expanded until the number of cells was enough for exosome production.

### Preparation of Exosomes

When the density reached 80%, cells in four 10‐cm dishes were washed with DPBS twice and the medium was exchanged with DMEM/High glucose supplemented with 10% exosome‐depleted FBS and 1% penicillin/streptomycin solution. After 24 h, the supernatant was collected for isolation of exosomes. Then, ultracentrifugation was used to obtain exosomes. First, the supernatant was centrifuged at 300 g for 10 min in order to remove the dead cells. Second, the supernatant transferred into a new centrifuge tube was centrifuged at 2000 g for 10 min in order to remove cell debris. Third, the supernatant transferred into a new centrifuge tube was centrifuged at 10 000 g for 10 min to discard microvesicles. Fourth, the supernatant transferred into a new centrifuge tube was filtered with a 0.22 µm filter (Steritop; Millipore) to remove apoptotic bodies. Fifth, the supernatant was centrifuged at 1 00 000 g for 90 min to obtain exosomes. Lastly, the exosomes were treated with RIPA supplemented with a protease inhibitor or resuspended with 200 µl DPBS and stored at −80 °C until use.

### Exosome Identification

The characteristics of exosomes were evaluated using four approaches, including Nanosight, transmission electron microscope (TEM), Western blot, flow cytometer (ImageStreamX Mark II). For Nansight, the exosomes adequately resuspended with 1 ml PBS were gently added into Nanosight NS300 (Malvern) sample pool. Next, put the laser module back on the pedestal and place a probe with OMEGA thermometer in Copper hole of laser module. Then adjust the suitable screen gain and camera level. When all parameters were ready, measured the samples. Finally, the samples and particle concentration (particles/ml) was counted. For TEM, place 5–10 µl exosomes on the copper screen and settle for 3 min. Next, remove the liquid near the edge by using filter paper. Then, negatively stained with phosphotungstic acid after *PBS* rinsed. Finally, exsomes were dried at room temperature and examined by TEM (Japan, JEM‐1200EX). Western blot was performed according to the previous studies.^[^ ^38^ ^]^ The antibodies were mouse anti‐lamp2 antibody, rabbit anti‐TSG101, rabbit anti‐CD63, rabbit anti‐CD81, and mouse anti‐β‐actin. For the flow cytometer, it could detect the exosomes with GFP due to the insertion of fusion protein LAMP2B. Exosomes were isolated from supernatant and measured by amnis ImageStream, in which the 100 nm FITC particles were used as the standard control.

### RT‐PCR

At the end of cell treatment, the total RNA was extracted with Total RNA Extraction Kit (MAJIOIVD, Magnetic Bead Method) according to the manufacturer's instructions. Next, complementary DNA sequences were synthesized by using PrimeScriptTM RT reagent kit with gDNA eraser (Takara, Japan) according to the manufacturer's protocol. Then, RT‐PCR was operated on Mx3000 PCR machine (Stratagene) by SYBR Premix Ex Taq II kit (Takara, RR820A). The following primer sets for qPCR were used: HIF‐1α,5^′^‐GGGGAGGACGATGAACATCAA‐3^′^, complementary sequence 5′‐GGGTGGTTTCTTGTACCCACA3’;Cyclophilin,5′CGAGCTCTGAGCACTGGAGA‐3′; complementary sequence, 5′‐ TGGCGTGTAA AGTCACCACC‐3′.

### The Separation of Primary Cells and Cell Culture

The primary chondrocytes were separated from murine articular cartilage.^[^ ^39^ ^]^ The following steps have been done in sterile environment: 1) obtained femoral heads, femoral condyles and tibial plateau after removing skin and soft tissues. 2) Placed them in 1 × DPBS and washed twice with 1 × DPBS. 3) Discarded DPBS and put them into centrifuge tube with 10 ml 0.1% type II collagenase at 37 °C in an incubator under 5%CO_2_ overnight. 4) The tube was centrifuged at 1000 rpm for 5 min. 5) Discarded the supernatant and seed primary chondrocytes in culture dishes with DMEM/F12 supplemented with 10% FBS.

The primary fibroblasts were separated from mouse synovium. The following steps have been done in sterile environment: 1) obtained synovium near the knee joint as much as possible. 2) Cut the synovium into 1 mm^3^ pieces and digested them with type IV collagenase overnight in 15 ml centrifuge tube placed in shaker at 37 °C. 3) Centrifuged at 1000 rpm for 5 min. 4) Discarded the supernatant and seed the cells in culture dishes.

The bone marrow‐derived macrophages (BMDM) were obtained from bone marrow of adult mice^[^ ^40^ ^]^ using the following steps in sterile environment: 1) Obtained femur and tibia after removing the muscle around them. 2) Cut base and knee joint of femur and epiphyses of tibia to obtain bone marrow through a 23G needle. 3) Washed the bone marrow with 2, 3 ml DMEM/High glucose supplemented with 10% FBS in 10 cm dishes. 4) Transferred the supernatant into 15 ml centrifuge tube and centrifuged at 200 g for 5 min under 4 °C. 5) Discarded the supernatant and resuspended sediment with complete medium. 6) Seed cells in 10 cm dishes. 7) Collected the supernatant in 10 cm dishes after 24 h and centrifuge at 1000 rpm. 8) Resuspended the sediment with complete medium and seed in 6‐well plates supplemented with 10 ng ml^−1^ M‐CSF. 9) Changed the medium with fresh complete medium supplemented with 10 ng ml^−1^ M‐CSF at day 3. 10) Evaluated the formation of BMDM. In order to polarize BMDM into pro‐inflammatory macrophages, 100 ng ml^−1^ LPS and 50 ng ml^−1^ INF‐γwere added in complete medium and incubated for 24 h. To polarize BMDM into anti‐inflammatory macrophages, 10 ng ml^−1^ IL‐4 was added in complete medium and incubated for 24 h.

As previous study reported,^[^ ^2b^ ^]^ the primary peritoneal macrophages were isolated using the following steps: 1) Surgical instruments (scissors, forceps, etc.) were sterilized, and the dissection table was disinfected with ultraviolet light. 2) Mice were euthanized by cervical dislocation and immersed in 75% ethanol for 5 min to ensure disinfection. 3) The abdominal skin was gently lifted using forceps, a small incision was made, and the abdominal wall was carefully dissected to expose the peritoneum below the xiphoid process, ensuring the integrity of the peritoneal membrane. 4) A 10 mL syringe was used to aspirate 1640 medium containing 5% FBS (pre‐cooled on ice), and it was carefully injected into the abdominal cavity above the liver to prevent accidental penetration of the intestines. 5) ≈5, 6 mL of medium was gently infused into the abdominal cavity, followed by a 15 min abdominal massage to ensure thorough distribution. 6) The liquid from the abdominal cavity was aspirated using a syringe and transferred to a 15 mL centrifuge tube, then centrifuged at 1000 rpm for 5 min. 7) The collected cells were suspended in 1640 medium supplemented with 10% FBS and 1% antibiotics, and incubated at 37 °C for 2–4 h. 8) After incubation, the cells were washed with PBS to remove non‐adherent cells, and the remaining adherent cells were considered peritoneal macrophages (with a purity of 85%–95%).

For RAW 264.7 cell line, incubated with 100 ng ml^−1^ LPS for 24 h to generate pro‐inflammatory macrophages or incubated with 20 ng ml^−1^ IL‐4 to obtain anti‐inflammatory macrophages.

### The Inhibition of Pathways Participating in Exosome Uptake by Cells

To generate pro‐inflammatory macrophages, RAW 264.7 cells were incubated with 100 ng ml^−1^ LPS for 24 h. Then, for phagocytosis and macropinocytosis, Wortmannin (1 µm) and Amiloride (1 mm) were used to treat pro‐inflammatory macrophages for 30 min.^[^ ^41^ ^]^ For clathrin‐mediated and caveolin‐mediated endocytosis, chlorpromazine (10 µg ml^−1^) was used to treat macrophages for 30 min, while the dynasore (80 µg ml^−1^) was used to treat macrophages for 1 h.^[^ ^42^ ^]^ Subsequently, AnCar‐Exo^LaIMTS3^ was added for co‐incubation for 12 h. Finally, immunofluorescence was performed to observe the enrichment level of AnCar‐Exo^LaIMTS3^ in pro‐inflammatory macrophages.

### Flow Cytometry

The BMDM was seeded in 35 mm‐confocal dishes at the density of 2 × 105 cells with complete medium containing nothing or 100 ng ml^−1^ LPS and 50 ng ml^−1^ INF‐γ or 10 ng ml^−1^ IL‐4 for 24 h. The complete medium was then replaced with complete medium containing 10% exosome‐depleted FBS and incubated with 1 × 10^8^ AnCar‐Exo^LaIMTS3^ for 12 h at 37 °C. Later, Collected the Mφ, pro‐inflammatory and anti‐inflammatory macrophages and resuspended them with FACS buffer (PBS containing 1% FBS, 1 mM EDTA). Next, prepared for blank control, and Isotype control for the following analysis. Incubated with antibodies against F4/80, CD86, CD206 (1:100, Biolegend) at 4 °C for 30 min. Then, washed with FACS buffer. The cells were centrifuged at 500 g for 5 min. At last, over 50 000 events were collected for analyzing by Flow cytometry (Beckman, CytoFLEX SRT).

### Exosome Tracing In Vivo

Exosomes were characterized in mice following systemic or local administration of them. Arthritis was induced by injecting collagenase into knee joint. Exosomes were labeled with DID. For systemic administration, mice were randomly divided into three groups. After injected collagenase at day 7, the arthritis mice were injected with exosomes (1 × 10^9^ particles per mouse) according to the dose of previous study.^[^ ^43^ ^]^ Then, the mice imaging was detected by Fusion FX Edge system (Vilber Lourmat). The signal intensity was recorded after injecting exosomes at 0, 1, 3, 6, 12, 24, and 48 h. Three hours after injection, three mice were sacrificed to observe the distribution of exosomes in major organs. For local administration in particular cavities, mice were also randomly divided into three groups. Seven days after collagenase injection, the arthritic mice were injected with exosomes (1 × 10^9^ particles per mouse). Mice were then imaged by Fusion FX Edge system. The signal intensity was recorded after injecting exosomes at 0, 1, 3, 6, 12, 24, 48, and 72 h. The exposure time, focus and position of mice were the same between mice with local or systemic administration. The FusionCapt Advance software was used to analyze the images. Briefly, the interesting regions showing signal intensity were identified. Then, the background of images was balanced and original intensities of fluorescence were recorded. Last, the real intensities of exosomes were obtained by subtracting their background fluorescence from the original intensities.

For *tdTomato‐ LysM^Cre^* mice, the mice were injected with 1 × 10^9^ particles into a vail vein after being placed in 10cm‐dishes containing 3% low‐melting hydrogel to immobilize the mouse. Then mice were detected by Two‐Photon Microscopy from 0 min to 120 min.

For *Mpeg1:GAL4/UAS:NTR‐mCherry* zebrafish which was used to trace macrophage in vivo.^[^ ^11^, ^16^ ^]^ First, an injury in the zebrafish caudal fin was induced using a surgical scalpel to create the injury model. After 7 hours, the zebrafish were anesthetized and fixed. Subsequently, a microinjector was used to administer exosomes into the zebrafish heart region. Finally, the injured portion of the zebrafish caudal fin was positioned under a two‐photon microscope for observation.

### Arthritis Induction and Exosome Injection

Twelve‐weeks‐old’ C57BL/6J male mice (Charles River) were divided into four groups (5 males/group). The approaches to induce arthritis were described in previous study.^[^ ^43^ ^]^ After being anesthetized with 0.8% pentobarbital, the mice in sham group had the skin of the right knee joint cut without injecting collagenase. While for arthritis group, the knee joints were fully exposed and injected with 1 unit type VII collagenase (Sigma, C0773) in 10 µl CaCl_2_ at day 1 and day 3, respectively. For transgenic mice, the same procedures were applied to experimental groups.

The cargoes for AnCar‐Exo^LaIMTS3^ were loaded by using Exo‐Fect exosome transfection reagent (SBI, EXFT20A‐1). In brief, ≈1 × 10^9^ exosomes were transfected with 20 pmol siRNA using 10 µl Exo‐Fect exosome transfection reagent. After incubating the exosome transfection solution at 37 °C in a shaker for 10 min, the mixture was placed on ice immediately. Exosomes were injected into joint cavity at the dose of 1 × 10^9^ particles per mouse. According to the distribution of exosomes in vivo, exosomes were injected at days 4, 7, 10, and 13 after induction of arthritis by collagenase at day 1. Mice were sacrificed on day 14. The sequences of siRNA‐HIF‐1α were the following: siRNA‐HIF‐1α−1, GGAAAGAACUAAACACACATT; siRNA‐HIF‐1α−2, CUGAAGACACAGAGGCAAATT; siRNA‐HIF‐1α−3, AAACAGAGACGAAGGACAATT.

### Gait Analysis

Before formal gait analysis experiment, all mice were trained to be familiar with the test environment and can cross the walkway without interruptions or hitches in video‐based CatWalk gait analysis system (Netherlands, Noldus). The system was composed of an enclosed walkway, a yellow fluorescent lamp, a high‐speed color camera and analysis software. The gait data were recorded by a high‐speed color camera.

### X‐Ray and MRI

All mice were examined by X‐ray using Faxitron MX‐20 (Faxitron Bioptics, Tucson USA) at 26 kV. For MRI, the mice were anesthetized with 0.8% pentobarbital and scanned by a small animal MRI (Germany, Bruker BioSpec USR 70/20). After the right leg knee joint was fixed with four channels coil, the mice were scanned with T1WI, T2WI. The scanning parameters are: 1) T1WI(RARE sequence): RARE factor = 4, TR = 693.0 ms, TE = 6.0 ms, Flip angle = 90°, FOV = 25 mm × 25 mm, Matrix = 256 × 256, layer thickness = 0.4 mm, Number of layer = 8, scanning time = 10 min. 2) T2WI (Turbo RARE sequence):RARE factor = 6, TR = 3000 ms, TE = 45 ms, Flip angle = 90°, FOV = 25 mm × 25 mm, Matrix = 256 × 256, layer thickness = 0.4 mm, Number of layer = 8, scanning time = 10 min.

### Histology Analysis

All samples of knee joints went through 4% paraformaldehyde fixation, 0.5 m EDTA at pH 7.4, 30% sucrose and optimal cutting temperature (OCT) compound embedding. The OCT sections (10 µm thickness) were chosen for Immunofluorescence as described in the previous study.^[^ ^14a^ ^]^ After being blocked with QuickBlock Blocking Buffer for Immunol Staining (Beyotime, P0260), those sections were incubated at 4 °C overnight with the primary antibody such as F4/80, iNOS, CD206. Then sections were incubated with secondary antibodies conjugated with fluorescence (1:500; Invitrogen/Abcam) at 37 °C for 1 h. Last, DAPI (1:1000; D8417, Sigma‐Aldrich) was used to stain the nucleus. Samples were detected by the living cells workstation (Olympus, Tokyo, Japan).

### Bioinformatics Analysis of Proteins Containing the Ancar‐Laimts3 Amino Acid Sequence

To explore proteins containing the AnCar‐LaIMTS3 amino acid sequence, a comprehensive search was conducted through the NCBI protein database. The focus was on identifying proteins with sequences aligned to AnCar‐LaIMTS3. Initially, a protein BLAST by inputting the AnCar‐LaIMTS3 sequence into the “Enter Query Sequence” field was employed. The search by setting the organism as Mus and selecting “protein‐protein blast” as the program was narrowed down. Subsequently, a range of proteins containing this sequence was identified. The selection by considering the Max score and E value was further refined. This process led to shortlisting the top 100 ranked proteins.

### Immunofluorescence

The OCT sections (10 µm thickness) were chosen for Immunofluorescence. All samples of knee joint sequentially went through 4% paraformaldehyde fixation, 0.5 m EDTA at pH 7.4, 30% sucrose and optimal cutting temperature (OCT) compound embedding. The Immunofluorescence procedures were described as previous study.^[^ ^14a^ ^]^ After being blocked with QuickBlock Blocking Buffer for Immunol Staining (Beyotime, P0260), sections were incubated at 4 °C overnight with the primary antibody such as F4/80, iNOS, CD206. Sections were then incubated with secondary antibodies conjugated with fluorescence (1:500; Invitrogen/Abcam) at 37 °C for 1 h. Last, DAPI (1:1000; D8417, Sigma‐Aldrich) was used to stain the nucleus. Signals in the section were detected by the living cells workstation (Olympus, Tokyo, Japan).

### Statistical analysis

All values were displayed as the way of mean ± SD. One‐way or two‐way analysis of variance (ANOVA) with a Tukey post‐hoc test was used to evaluate the difference among groups. P < 0.05 were considered statistically significant (*P < 0.05, **P < 0.01, ***P < 0.005, and ****P < 0.001). The software for Statistical analysis was Graph Pad Prism 9.0 (GraphPad Software Inc., La Jolla, CA, USA).

## Conflict of Interest

The authors declare no conflict of interest.

## Author contributions

S.L. was responsible for the majority of experiments and finished the manuscript. Y.R.W., X.Q.P., H.B.Q. and X.W., participated in the experiments and helped with manuscript. H.G.C., H.C., J.Y., Y.H., T.Q.Y., X.L.D., and Y.L.X. improved the manuscript. J.L.H., R.B.Z, L.X.H., H.B., P.Y., and F.T.L. helped with mice housing and genotype identification. M.J., N.S., X.Q.L., S.H., Y.X.J., T.Y.Z, S.R.Z., Y.X and Y.Z. revised the manuscript. Y.Y.L. and G.Y.X helped with two photo microscope. L.C., Z.H.N. and L.K. were responsible for developing the study, supervising the project, conceiving the experiments, analyzing the data and critically reviewing the manuscript.

## Supporting information

Supporting Information

Supporting Information

Supplemental Movie 1

Supplemental Movie 2

## Data Availability

The data that support the findings of this study are available in the supplementary material of this article.
