# Supplementary material for: A Novel Cargo Delivery System‐AnCar‐ExoLaIMTS Ameliorates Arthritis via Specifically Targeting Pro‐Inflammatory Macrophages
Source: Adv Sci (Weinh). 2023 Dec 11;11(7):2306143. doi: 10.1002/advs.202306143 (PMC10870055; doi:10.1002/advs.202306143)
Supplement: Supplementary file 1 — Supporting Information [file ADVS-11-2306143-s002.pdf]

ADVANCED SCIENCE
Open Access

Supporting Information

for *Adv. Sci.*, DOI 10.1002/advs.202306143

A Novel Cargo Delivery System-AnCar-Exo^{LaIMTS} Ameliorates Arthritis via Specifically Targeting Pro-Inflammatory Macrophages

Song Li, Ya-ran Wu, Xiu-qin Peng, Han-gang Chen, Tong-yi Zhang, Hua Chen, Jing Yang, Yang-li Xie, Hua-bing Qi, Wei Xiang, Bo Huang, Si-ru Zhou, Yan Hu, Qiao-yan Tan, Xiao-lan Du, Jun-lan Huang, Ruo-bin Zhang, Xiao-hong Li, Feng-tao Luo, Min Jin, Nan Su, Xiao-qing Luo, Shuo Huang, Peng Yang, Xiao-Jing Yan, Ji-qin Lian, Ying Zhu, Yan Xiong, Gong-yi Xiao, Ying-ying Liu, Chen Shen, Liang Kuang, Zhen-hong Ni* and Lin Chen**

Supporting Information

A novel cargo delivery system- AnCar-Exo^{LaIMTS} ameliorates arthritis via specifically targeting pro-inflammatory macrophages

Song Li, Ya-ran Wu, Xiu-qin Peng, Han-gang Chen, Tong-yi Zhang, Hua Chen, Jing Yang, Yang-li Xie, Hua-bing Qi, Wei Xiang, Bo Huang, Si-ru Zhou, Yan Hu, Qiao-yan Tan, Xiao-lan Du, Jun-lan Huang, Ruo-bin Zhang, Xiao-hong Li, Feng-tao Luo, Min Jin, Nan Su, Xiao-qing Luo, Shuo Huang, Peng Yang, Xiao-Jing Yan, Ji-qin Lian, Ying Zhu, Yan Xiong, Gong-yi Xiao, Ying-ying Liu, Chen Shen, Liang Kuang, Zhen-hong Ni*, Lin Chen*.*

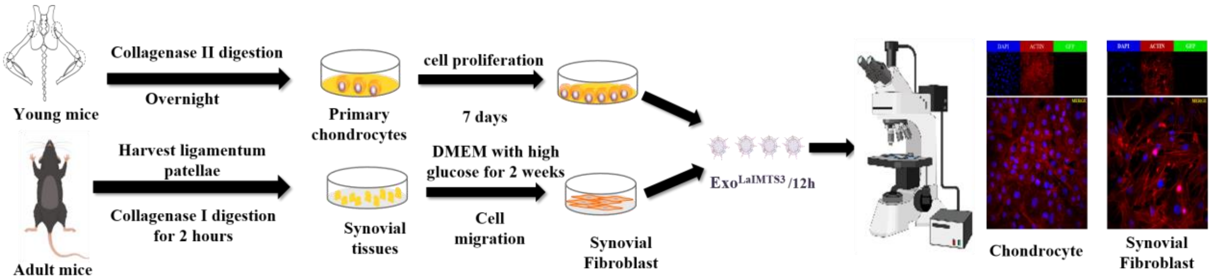

Figure. S1. The uptake of AnCar-Exo^{LaIMTS3} by primary chondrocyte and synovial fibroblast. Immunofluorescence showed ACTIN (red) and GFP (green) in exosome-treated chondrocyte and synovial fibroblast.

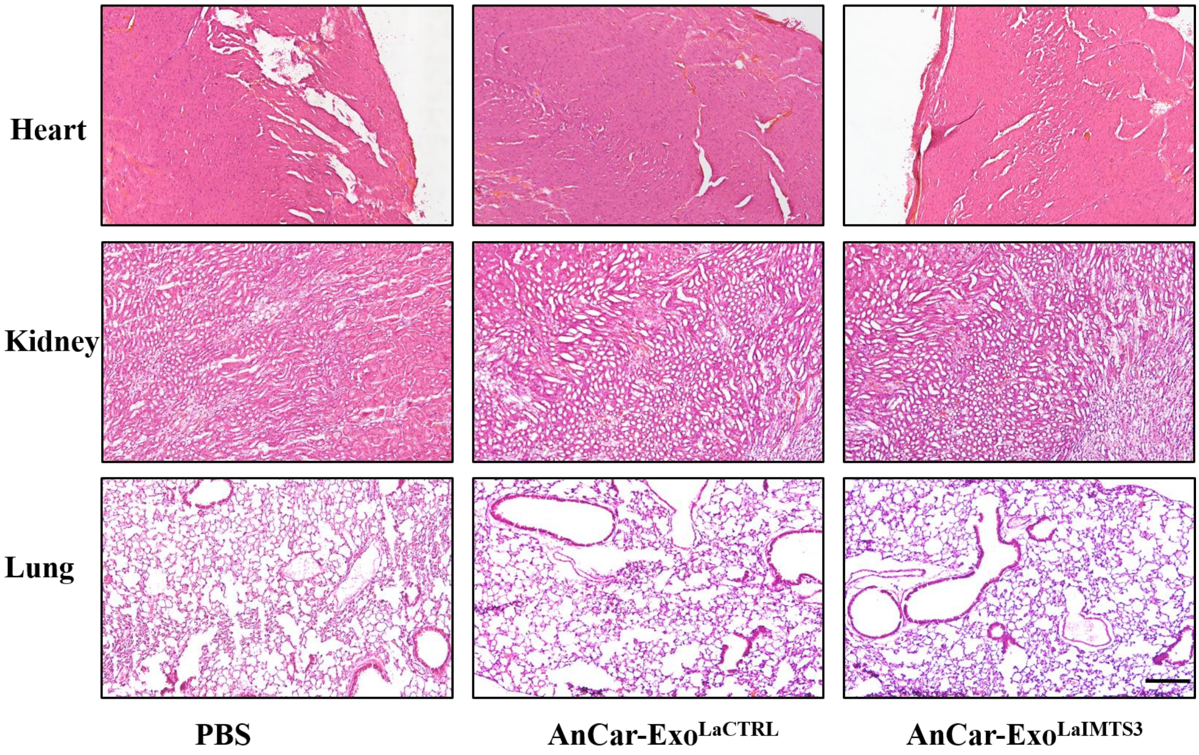

Figure. S2. Hematoxylin-eosin staining of heart, kidney and lung at 48h after injection. N=3 per group. Scale bars, 100 μm.

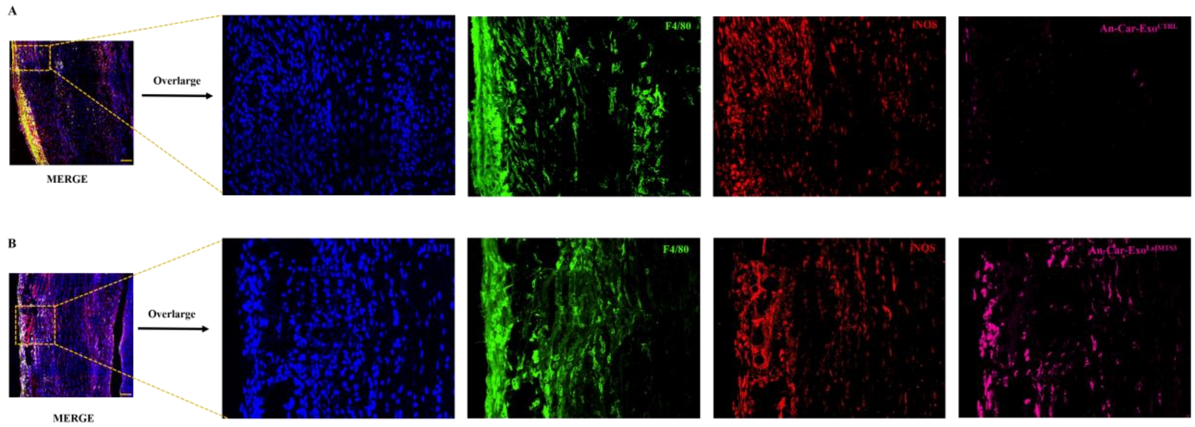

Figure. S3. AnCar-Exo^{LaIMTS3} was mainly enriched in pro-inflammatory macrophage in synovial tissues of inflammatory joint after systemic administration. A) Immunofluorescence show F4/80 (green), iNOS (red) and DID (pink) after intra-articular injection with AnCar-Exo^{LaCTRL}. DID stands for exosome signal. Scale bars, 200 μm. N=5 per group. B) Immunofluorescence show F4/80 (green), iNOS (red) and DID (pink) after intra-articular injection with AnCar-Exo^{LaIMTS3}. DID stands for exosome signal. Scale bars, 200 μm. N=5 per group.

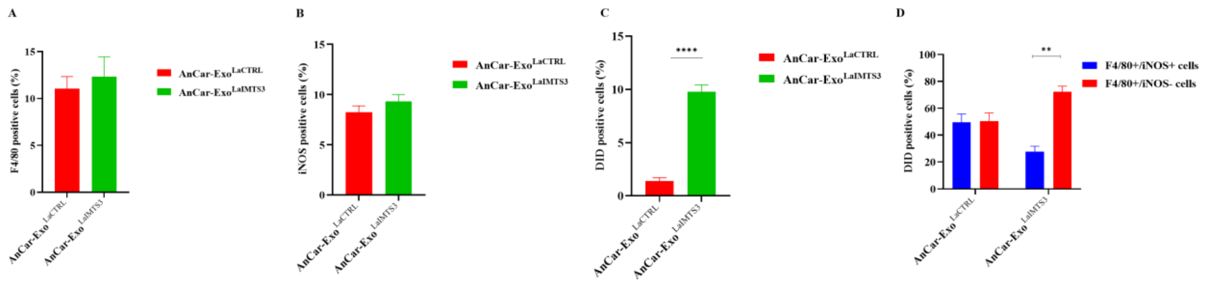

Figure. S4. AnCar-Exo^{LaIMTST3} was mainly enriched in pro-inflammatory macrophage in synovial tissues of inflammatory joint after systemic administration. A) Quantification of F4/80 positive cells between AnCar-Exo^{LaCTRL} and AnCar-Exo^{LaIMTS3} groups. N=5 per group. B) Quantification of iNOS positive cells between AnCar-Exo^{LaCTRL} and AnCar-Exo^{LaIMTS3} groups. N=5 per group. C) Quantification of DID positive cells between AnCar-Exo^{LaCTRL} and AnCar-Exo^{LaIMTS3} groups. N=5 per group, ****$P < 0.0001$. D) Quantification of DID positive cells in F4/80^{+} iNOS^{+} cells and F4/80^{+} iNOS^{-} cells between AnCar-Exo^{LaCTRL} and AnCar-Exo^{LaIMTS3} groups. N=5 per group, **$P < 0.01$. All values were displayed as the way of mean

± SD. One-way analysis of variance (ANOVA) was used to evaluate the difference among groups.

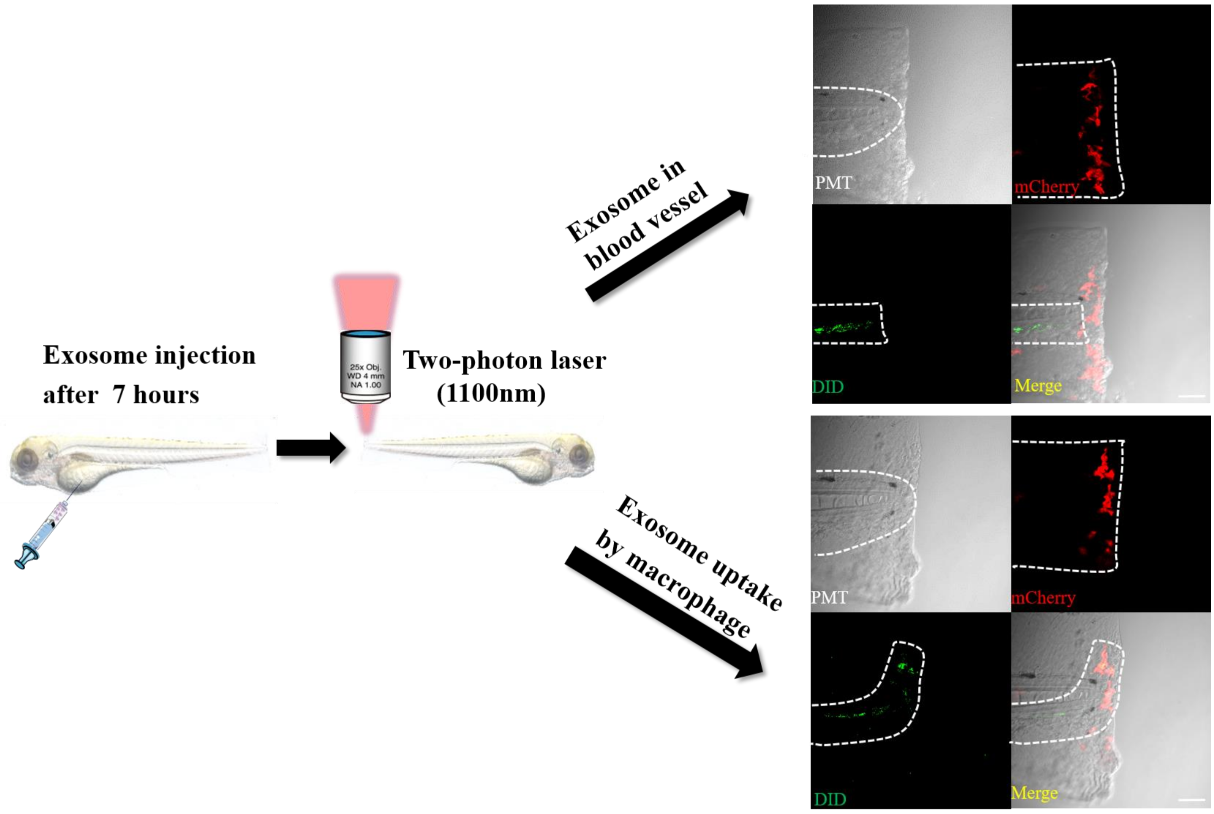

Figure. S5. Exosomes enter blood vessels from the heart and eventually reach the caudal fin.

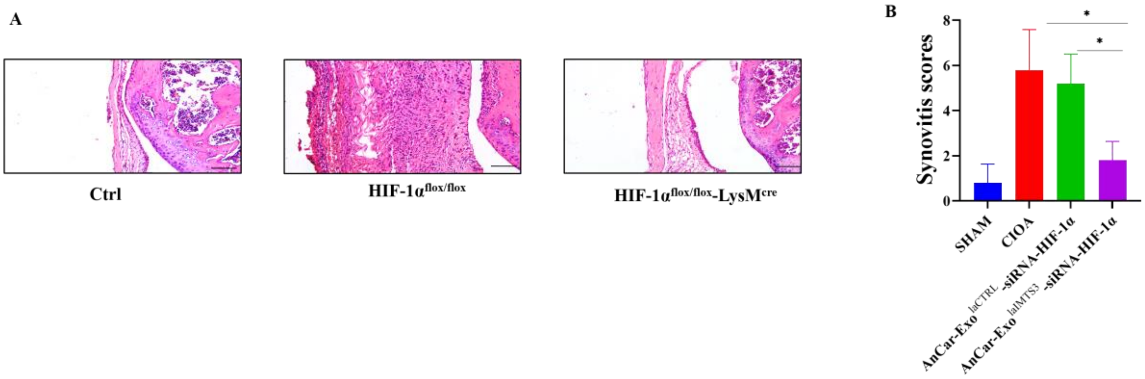

Figure. S6. Synovitis was shown by H&E staining and Synovitis scores. All values were displayed as the way of mean ± SD. One-way analysis of variance (ANOVA) was used to evaluate the difference among groups. N=5 per group, *P <0.05.

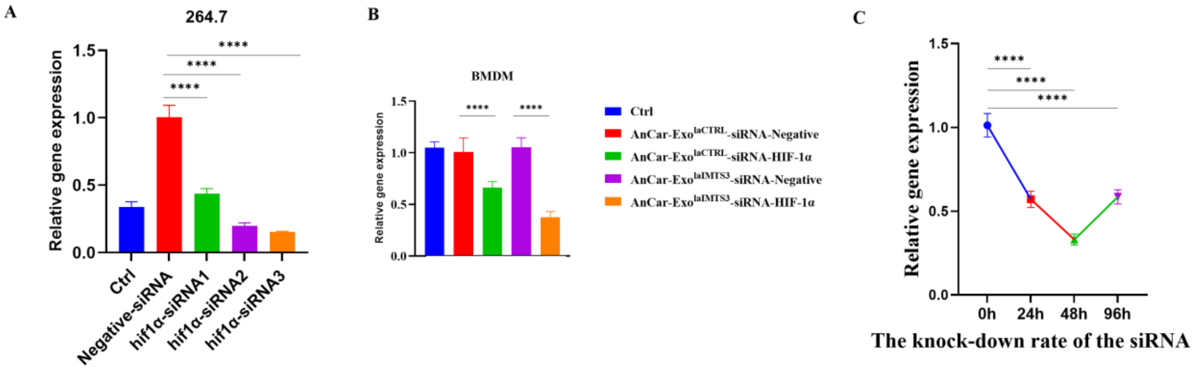

Figure. S7. A and B) The inhibition efficiency of siRNA-HIF-1α in LPS-activated RAW 264.7 or LPS/IFN-γ activated BMDM. N=3 independent biological replicates, ****P<0.0001. C) The knock-down rate of the HIF-1α-siRNA loaded in AnCar-Exo^{LaIMTST3} after pro-inflammatory macrophages were treated for 0h,24h,48h,96h. N=3 independent biological replicates. ****P<0.0001. All values were displayed as the way of mean ± SD. One-way analysis of variance (ANOVA) was used to evaluate the difference among groups.

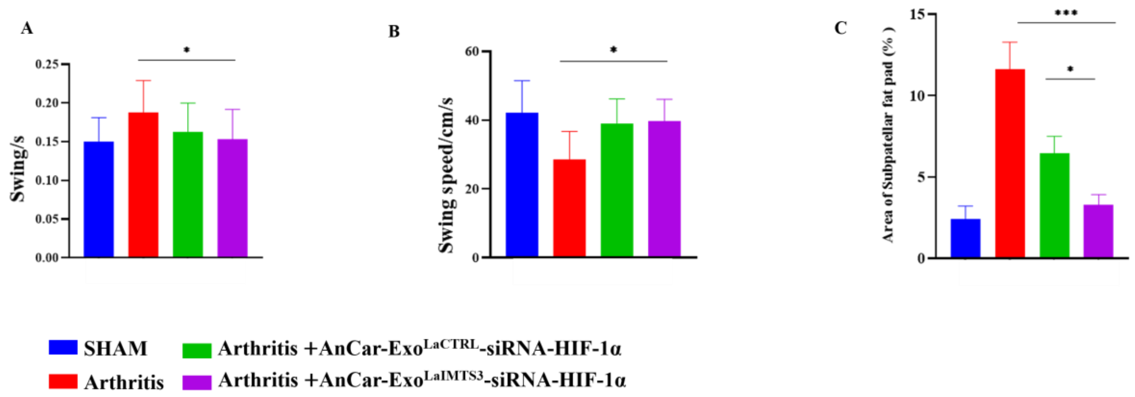

Figure. S8. The changes of gait parameters among groups (n=5 per group), including Swing, Swing speed and Quantification analysis of the area of Subpatellar fat pad. *P < 0.05, ***P < 0.001. All values were displayed as the way of mean ± SD. One-way analysis of variance (ANOVA) was used to evaluate the difference among groups.

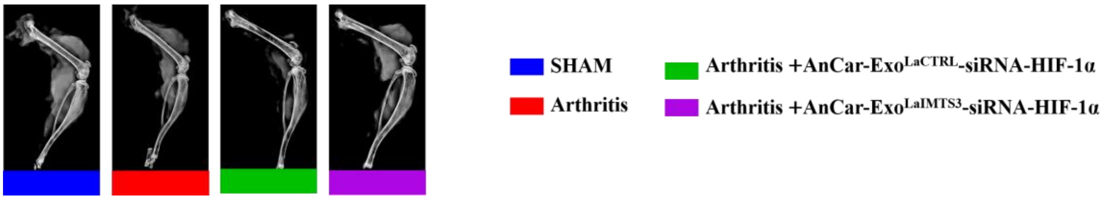

Figure. S9. X ray images of right legs after two weeks' treatment among groups.

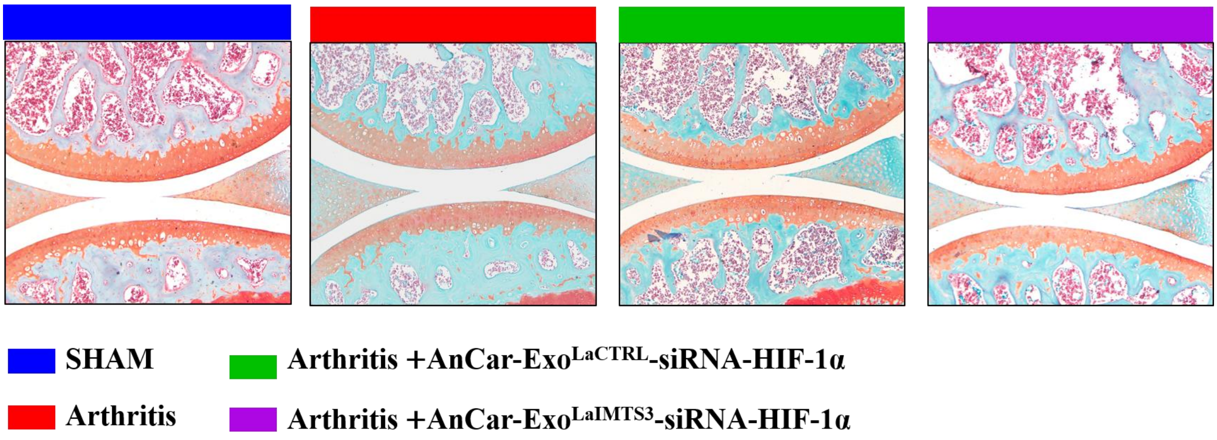

Figure. S10. Representative images of cartilage among groups after two weeks' treatment among groups.

Table S1. Gene sequence of AnCar-LaIMT plasmids

Plasmid	Gene Sequence
AnCar-LaIMT1	ATGGTGTGCTTCCGCCTCTTCCCGGTTCCGGGCTCAGGGCTCGTTCTGGTCTGCCTAG TCCTGGGAGCTGTGCGGTCTTATGCAGGAGGAGGAGGATCTTTTCAGCACCCTAGCT TTATCGGAGGGGGCGGGAGCTTTCAGCACCCCAGTTTTATCGGGGGAGGCGGGAG TTTCCAGCATCCCAGCTTTATTGGAGGCGGGGGCAGCTTGGAACTTAATTTGACAGA TTCAGAAAATGCCACTTGCCTTTATGCAAAATGGCAGATGAATTTCACAGTACGCTAT GAAACTACAAATAAAACTTATAAAACTGTAACCATTTCAGACCATGGCACTGTGACATA TAATGGAAGCATTTGTGGGGATGATCAGAATGGTCCCAAAATAGCAGTGCAGTTCGG ACCTGGCTTTTCCTGGATTGCGAATTTTACCAAGGCAGCATCTACTTATTCAATTGACA GCGTCTCATTTTCCTACAACACTGGTGATAACACAACATTTCCTGATGCTGAAGATAAA GGAATTCTTACTGTTGATGAACTTTTGGCCATCAGAATTCCATTGAATGACCTTTTTAG ATGCAATAGTTTATCAACTTTGGAAAAGAATGATGTTGTCCAACACTACTGGGATGTT CTTGTACAAGCTTTTGTCCAAAATGGCACAGTGAGCACAAATGAGTTCCTGTGTGATA AAGACAAAACTTCAACAGTGGCACCCACCATACACACCACTGTGCCATCTCCTACTAC AACACCTACTCCAAAGGAAAAACCAGAAGCTGGAACCTATTCAGTTAATAATGGCAA TGATACTTGTCTGCTGGCTACCATGGGGCTGCAGCTGAACATCACTCAGGATAAGGTT GCTTCAGTTATTAACATCAACCCCAATACAACTCACTCCACAGGCAGCTGCCGTTCTC ACACTGCTCTACTTAGACTCAATAGCAGCACCATTAAGTATCTAGACTTTGTCTTTGCT GTGAAAAATGAAAACCGATTTTATCTGAAGGAAGTGAACATCAGCATGTATTTGGTTA ATGGCTCCGTTTTCAGCATTGCAAATAACAATCTCAGCTACTGGGATGCCCCCCTGGG AAGTTCTTATATGTGCAACAAAGAGCAGACTGTTTCAGTGTCTGGAGCATTTCAGATA AATACCTTTGATCTAAGGGTTCAGCCTTTCAATGTGACACAAGGAAAGTATTCTACAG CCCAAGAGTGTTCGCTGGATGATGACACCATTCTAATCCCAATTATAGTTGGTGCTGG TCTTTCAGGCTTGATTATCGTTATAGTGATTGCTTACGTAATTGGCAGAAGAAAAAGTT ATGCTGGATATCAGACTCTG
AnCar-LaIMT2	GGAGGAGGAGGAAGCAGCCCACTGAGTCAGAGTGCAGGGGGGGGGGGAAGCAG CCCCCTGAGCCAGAGCGCTGGAGGAGGAGGCAGCAGCCCTCTGAGCCAGAGTGCA GGAGGAGGAGGGAGCTTGGAACTTAATTTGACAGATTCAGAAAATGCCACTTGCCT TTATGCAAAATGGCAGATGAATTTCACAGTACGCTATGAAACTACAAATAAAACTTATA AAACTGTAACCATTTCAGACCATGGCACTGTGACATATAATGGAAGCATTTGTGGGGA

	TGATCAGAATGGTCCCAAAATAGCAGTGCAGTTCGGACCTGGCTTTTCCTGGATTGC<br>GAATTTTACCAAGGCAGCATCTACTTATTCAATTGACAGCGTCTCATTTTCCTACAACA<br>CTGGTGATAACACAACATTTCCTGATGCTGAAGATAAAGGAATTCTTACTGTTGATGA<br>ACTTTTGGCCATCAGAATTCCATTGAATGACCTTTTTAGATGCAATAGTTTATCAACTT<br>TGGAAAAGAATGATGTTGTCCAACACTACTGGGATGTTCTTGTACAAGCTTTTGTCCA<br>AAATGGCACAGTGAGCACAAATGAGTTCCTGTGTGATAAAGACAAAACTTCAACAGT<br>GGCACCCACCATACACACCACTGTGCCATCTCCTACTACAACACCTACTCCAAAGGAA<br>AAACCAGAAGCTGGAACCTATTCAGTTAATAATGGCAATGATACTTGTCTGCTGGCTA<br>CCATGGGGCTGCAGCTGAACATCACTCAGGATAAGGTTGCTTCAGTTATTAACATCAA<br>CCCCAATACAACTCACTCCACAGGCAGCTGCCGTTCTCACACTGCTCTACTTAGACTC<br>AATAGCAGCACCATTAAGTATCTAGACTTTGTCTTTGCTGTGAAAAATGAAAACCGAT<br>TTTATCTGAAGGAAGTGAACATCAGCATGTATTTGGTTAATGGCTCCGTTTTCAGCATT<br>GCAAATAACAATCTCAGCTACTGGGATGCCCCCCTGGGAAGTTCTTATATGTGCAACA<br>AAGAGCAGACTGTTTCAGTGTCTGGAGCATTTCAGATAAATACCTTTGATCTAAGGGT<br>TCAGCCTTTCAATGTGACACAAGGAAAGTATTCTACAGCCCAAGAGTGTTCGCTGGA<br>TGATGACACCATTCTAATCCCAATTATAGTTGGTGCTGGTCTTTCAGGCTTGATTATCG<br>TTATAGTGATTGCTTACGTAATTGGCAGAAGAAAAAGTTATGCTGGATATCAGACTCT<br>G
**AnCar-LaIMT3**	GGAGGAGGAGGAAGCCTGCCAAGCAGTGGAGCCGCCGGAGGAGGAGGGAGCCT<br>GCCTAGTAGTGGAGCCGCTGGAGGAGGAGGCAGCCTGCCAAGTAGCGGAGCCGCC<br>GGCGGAGGAGGAAGTTTGGAACTTAATTTGACAGATTCAGAAAATGCCACTTGCCT<br>TTATGCAAAATGGCAGATGAATTTCACAGTACGCTATGAAACTACAAATAAAACTTATA<br>AAACTGTAACCATTTCAGACCATGGCACTGTGACATATAATGGAAGCATTTGTGGGGA<br>TGATCAGAATGGTCCCAAAATAGCAGTGCAGTTCGGACCTGGCTTTTCCTGGATTGC<br>GAATTTTACCAAGGCAGCATCTACTTATTCAATTGACAGCGTCTCATTTTCCTACAACA<br>CTGGTGATAACACAACATTTCCTGATGCTGAAGATAAAGGAATTCTTACTGTTGATGA<br>ACTTTTGGCCATCAGAATTCCATTGAATGACCTTTTTAGATGCAATAGTTTATCAACTT<br>TGGAAAAGAATGATGTTGTCCAACACTACTGGGATGTTCTTGTACAAGCTTTTGTCCA<br>AAATGGCACAGTGAGCACAAATGAGTTCCTGTGTGATAAAGACAAAACTTCAACAGT<br>GGCACCCACCATACACACCACTGTGCCATCTCCTACTACAACACCTACTCCAAAGGAA<br>AAACCAGAAGCTGGAACCTATTCAGTTAATAATGGCAATGATACTTGTCTGCTGGCTA<br>CCATGGGGCTGCAGCTGAACATCACTCAGGATAAGGTTGCTTCAGTTATTAACATCAA<br>CCCCAATACAACTCACTCCACAGGCAGCTGCCGTTCTCACACTGCTCTACTTAGACTC<br>AATAGCAGCACCATTAAGTATCTAGACTTTGTCTTTGCTGTGAAAAATGAAAACCGAT<br>TTTATCTGAAGGAAGTGAACATCAGCATGTATTTGGTTAATGGCTCCGTTTTCAGCATT<br>GCAAATAACAATCTCAGCTACTGGGATGCCCCCCTGGGAAGTTCTTATATGTGCAACA<br>AAGAGCAGACTGTTTCAGTGTCTGGAGCATTTCAGATAAATACCTTTGATCTAAGGGT<br>TCAGCCTTTCAATGTGACACAAGGAAAGTATTCTACAGCCCAAGAGTGTTCGCTGGA<br>TGATGACACCATTCTAATCCCAATTATAGTTGGTGCTGGTCTTTCAGGCTTGATTATCG<br>TTATAGTGATTGCTTACGTAATTGGCAGAAGAAAAAGTTATGCTGGATATCAGACTCT<br>G
**AnCar-LaIMT4**	GGAGGAGGAGGAAGCACAGGAAGCCCAGAGCTGCATGGAGGGGGGGGGAGCAC<br>AGGAAGTCCAGAGCTGCACGGAGGAGGAGGGAGCACAGGGAGCCCAGAGCTGCA<br>CGGGGGAGGAGGAAGTTTGGAACTTAATTTGACAGATTCAGAAAATGCCACTTGCC<br>TTTATGCAAAATGGCAGATGAATTTCACAGTACGCTATGAAACTACAAATAAAACTTAT<br>AAAACTGTAACCATTTCAGACCATGGCACTGTGACATATAATGGAAGCATTTGTGGGG<br>ATGATCAGAATGGTCCCAAAATAGCAGTGCAGTTCGGACCTGGCTTTTCCTGGATTGC<br>GAATTTTACCAAGGCAGCATCTACTTATTCAATTGACAGCGTCTCATTTTCCTACAACA

	CTGGTGATAACACAACATTTCCTGATGCTGAAGATAAAGGAATTCTTACTGTTGATGA<br>ACTTTTGGCCATCAGAATTCCATTGAATGACCTTTTTAGATGCAATAGTTTATCAACTT<br>TGGAAAAGAATGATGTTGTCCAACACTACTGGGATGTTCTTGTACAAGCTTTTGTCCA<br>AAATGGCACAGTGAGCACAAATGAGTTCCTGTGTGATAAAGACAAAACTTCAACAGT<br>GGCACCCACCATACACACCACTGTGCCATCTCCTACTACAACACCTACTCCAAAGGAA<br>AAACCAGAAGCTGGAACCTATTCAGTTAATAATGGCAATGATACTTGTCTGCTGGCTA<br>CCATGGGGCTGCAGCTGAACATCACTCAGGATAAGGTTGCTTCAGTTATTAACATCAA<br>CCCCAATACAACTCACTCCACAGGCAGCTGCCGTTCTCACACTGCTCTACTTAGACTC<br>AATAGCAGCACCATTAAGTATCTAGACTTTGTCTTTGCTGTGAAAAATGAAAACCGAT<br>TTTATCTGAAGGAAGTGAACATCAGCATGTATTTGGTTAATGGCTCCGTTTTCAGCATT<br>GCAAATAACAATCTCAGCTACTGGGATGCCCCCCTGGGAAGTTCTTATATGTGCAACA<br>AAGAGCAGACTGTTTCAGTGTCTGGAGCATTTCAGATAAATACCTTTGATCTAAGGGT<br>TCAGCCTTTCAATGTGACACAAGGAAAGTATTCTACAGCCCAAGAGTGTTCGCTGGA<br>TGATGACACCATTCTAATCCCAATTATAGTTGGTGCTGGTCTTTCAGGCTTGATTATCG<br>TTATAGTGATTGCTTACGTAATTGGCAGAAGAAAAAGTTATGCTGGATATCAGACTCT<br>G
**AnCar-LaIMT5**	GGAGGAGGAGGAAGCCAGATGCCAGAGATGAAGGCCGGAGGAGGAGGGAGCCA<br>GATGCCCGAGATGAAGGCTGGAGGAGGAGGCAGCCAGATGCCTGAGATGAAGGCC<br>GGGGGAGGAGGAAGTTTGGAACTTAATTTGACAGATTCAGAAAATGCCACTTGCCT<br>TTATGCAAAATGGCAGATGAATTTCACAGTACGCTATGAAACTACAAATAAAACTTATA<br>AAACTGTAACCATTTCAGACCATGGCACTGTGACATATAATGGAAGCATTTGTGGGGA<br>TGATCAGAATGGTCCCAAAATAGCAGTGCAGTTCGGACCTGGCTTTTCCTGGATTGC<br>GAATTTTACCAAGGCAGCATCTACTTATTCAATTGACAGCGTCTCATTTTCCTACAACA<br>CTGGTGATAACACAACATTTCCTGATGCTGAAGATAAAGGAATTCTTACTGTTGATGA<br>ACTTTTGGCCATCAGAATTCCATTGAATGACCTTTTTAGATGCAATAGTTTATCAACTT<br>TGGAAAAGAATGATGTTGTCCAACACTACTGGGATGTTCTTGTACAAGCTTTTGTCCA<br>AAATGGCACAGTGAGCACAAATGAGTTCCTGTGTGATAAAGACAAAACTTCAACAGT<br>GGCACCCACCATACACACCACTGTGCCATCTCCTACTACAACACCTACTCCAAAGGAA<br>AAACCAGAAGCTGGAACCTATTCAGTTAATAATGGCAATGATACTTGTCTGCTGGCTA<br>CCATGGGGCTGCAGCTGAACATCACTCAGGATAAGGTTGCTTCAGTTATTAACATCAA<br>CCCCAATACAACTCACTCCACAGGCAGCTGCCGTTCTCACACTGCTCTACTTAGACTC<br>AATAGCAGCACCATTAAGTATCTAGACTTTGTCTTTGCTGTGAAAAATGAAAACCGAT<br>TTTATCTGAAGGAAGTGAACATCAGCATGTATTTGGTTAATGGCTCCGTTTTCAGCATT<br>GCAAATAACAATCTCAGCTACTGGGATGCCCCCCTGGGAAGTTCTTATATGTGCAACA<br>AAGAGCAGACTGTTTCAGTGTCTGGAGCATTTCAGATAAATACCTTTGATCTAAGGGT<br>TCAGCCTTTCAATGTGACACAAGGAAAGTATTCTACAGCCCAAGAGTGTTCGCTGGA<br>TGATGACACCATTCTAATCCCAATTATAGTTGGTGCTGGTCTTTCAGGCTTGATTATCG<br>TTATAGTGATTGCTTACGTAATTGGCAGAAGAAAAAGTTATGCTGGATATCAGACTCT<br>G
**AnCar-LaIMT6**	GGAGGAGGAGGAAGCAGACAGAGCCACCTGAAGGTGGGAGGAGGGGGGAGCAG<br>ACAGAGTCACCTGAAGGTCGGAGGAGGAGGGAGCAGACAGTCACACCTGAAGGTG<br>GGGGGAGGAGGAAGTTTGGAACTTAATTTGACAGATTCAGAAAATGCCACTTGCCT<br>TTATGCAAAATGGCAGATGAATTTCACAGTACGCTATGAAACTACAAATAAAACTTATA<br>AAACTGTAACCATTTCAGACCATGGCACTGTGACATATAATGGAAGCATTTGTGGGGA<br>TGATCAGAATGGTCCCAAAATAGCAGTGCAGTTCGGACCTGGCTTTTCCTGGATTGC<br>GAATTTTACCAAGGCAGCATCTACTTATTCAATTGACAGCGTCTCATTTTCCTACAACA<br>CTGGTGATAACACAACATTTCCTGATGCTGAAGATAAAGGAATTCTTACTGTTGATGA<br>ACTTTTGGCCATCAGAATTCCATTGAATGACCTTTTTAGATGCAATAGTTTATCAACTT

	TGGAAAAGAATGATGTTGTCCAACACTACTGGGATGTTCTTGTACAAGCTTTTGTCCA AAATGGCACAGTGAGCACAAATGAGTTCCTGTGTGATAAAGACAAAACTTCAACAGT GGCACCCACCATACACACCACTGTGCCATCTCCTACTACAACACCTACTCCAAAGGAA AAACCAGAAGCTGGAACCTATTCAGTTAATAATGGCAATGATACTTGTCTGCTGGCTA CCATGGGGCTGCAGCTGAACATCACTCAGGATAAGGTTGCTTCAGTTATTAACATCAA CCCCAATACAACTCACTCCACAGGCAGCTGCCGTTCTCACACTGCTCTACTTAGACTC AATAGCAGCACCATTAAGTATCTAGACTTTGTCTTTGCTGTGAAAAATGAAAACCGAT TTTATCTGAAGGAAGTGAACATCAGCATGTATTTGGTTAATGGCTCCGTTTTCAGCATT GCAAATAACAATCTCAGCTACTGGGATGCCCCCCTGGGAAGTTCTTATATGTGCAACA AAGAGCAGACTGTTTCAGTGTCTGGAGCATTTCAGATAAATACCTTTGATCTAAGGGT TCAGCCTTTCAATGTGACACAAGGAAAGTATTCTACAGCCCAAGAGTGTTCGCTGGA TGATGACACCATTCTAATCCCAATTATAGTTGGTGCTGGTCTTTCAGGCTTGATTATCG TTATAGTGATTGCTTACGTAATTGGCAGAAGAAAAAGTTATGCTGGATATCAGACTCT G
**AnCar-LaIMT7**	GGAGGAGGAGGAAGCAGCGGATACCCTAGACACTATGGAGGGGGGGGGAGCAGC GGATATCCCAGACACTATGGGGGGGGGGGGTCTAGCGGATACCCAAGACACTACGG AGGAGGGGGAAGCTTGGAACTTAATTTGACAGATTCAGAAAATGCCACTTGCCTTTA TGCAAAATGGCAGATGAATTTCACAGTACGCTATGAAACTACAAATAAAACTTATAAA ACTGTAACCATTTCAGACCATGGCACTGTGACATATAATGGAAGCATTTGTGGGGATG ATCAGAATGGTCCCAAAATAGCAGTGCAGTTCGGACCTGGCTTTTCCTGGATTGCGA ATTTTACCAAGGCAGCATCTACTTATTCAATTGACAGCGTCTCATTTTCCTACAACACT GGTGATAACACAACATTTCCTGATGCTGAAGATAAAGGAATTCTTACTGTTGATGAAC TTTTGGCCATCAGAATTCCATTGAATGACCTTTTTAGATGCAATAGTTTATCAACTTTG GAAAAGAATGATGTTGTCCAACACTACTGGGATGTTCTTGTACAAGCTTTTGTCCAAA ATGGCACAGTGAGCACAAATGAGTTCCTGTGTGATAAAGACAAAACTTCAACAGTGG CACCCACCATACACACCACTGTGCCATCTCCTACTACAACACCTACTCCAAAGGAAAA ACCAGAAGCTGGAACCTATTCAGTTAATAATGGCAATGATACTTGTCTGCTGGCTACC ATGGGGCTGCAGCTGAACATCACTCAGGATAAGGTTGCTTCAGTTATTAACATCAACC CCAATACAACTCACTCCACAGGCAGCTGCCGTTCTCACACTGCTCTACTTAGACTCAAT AGCAGCACCATTAAGTATCTAGACTTTGTCTTTGCTGTGAAAAATGAAAACCGATTTT ATCTGAAGGAAGTGAACATCAGCATGTATTTGGTTAATGGCTCCGTTTTCAGCATTGC AAATAACAATCTCAGCTACTGGGATGCCCCCCTGGGAAGTTCTTATATGTGCAACAAA GAGCAGACTGTTTCAGTGTCTGGAGCATTTCAGATAAATACCTTTGATCTAAGGGTTC AGCCTTTCAATGTGACACAAGGAAAGTATTCTACAGCCCAAGAGTGTTCGCTGGATG ATGACACCATTCTAATCCCAATTATAGTTGGTGCTGGTCTTTCAGGCTTGATTATCGTTA TAGTGATTGCTTACGTAATTGGCAGAAGAAAAAGTTATGCTGGATATCAGACTCTG
**AnCar-LaIMT8**	GGAGGAGGAGGAAGCAAGTACAACACACACCACGCCGGCGGAGGCGGAAGCAAA TACAACACACATCACGCTGGAGGAGGAGGGAGCAAGTATAACACACACCATGCTGG AGGGGGCGGAAGCTTGGAACTTAATTTGACAGATTCAGAAAATGCCACTTGCCTTTA TGCAAAATGGCAGATGAATTTCACAGTACGCTATGAAACTACAAATAAAACTTATAAA ACTGTAACCATTTCAGACCATGGCACTGTGACATATAATGGAAGCATTTGTGGGGATG ATCAGAATGGTCCCAAAATAGCAGTGCAGTTCGGACCTGGCTTTTCCTGGATTGCGA ATTTTACCAAGGCAGCATCTACTTATTCAATTGACAGCGTCTCATTTTCCTACAACACT GGTGATAACACAACATTTCCTGATGCTGAAGATAAAGGAATTCTTACTGTTGATGAAC TTTTGGCCATCAGAATTCCATTGAATGACCTTTTTAGATGCAATAGTTTATCAACTTTG GAAAAGAATGATGTTGTCCAACACTACTGGGATGTTCTTGTACAAGCTTTTGTCCAAA ATGGCACAGTGAGCACAAATGAGTTCCTGTGTGATAAAGACAAAACTTCAACAGTGG CACCCACCATACACACCACTGTGCCATCTCCTACTACAACACCTACTCCAAAGGAAAA

	ACCAGAAGCTGGAACCTATTCAGTTAATAATGGCAATGATACTTGTCTGCTGGCTACC<br>ATGGGGCTGCAGCTGAACATCACTCAGGATAAGGTTGCTTCAGTTATTAACATCAACC<br>CCAATACAACTCACTCCACAGGCAGCTGCCGTTCTCACACTGCTCTACTTAGACTCAAT<br>AGCAGCACCATTAAGTATCTAGACTTTGTCTTTGCTGTGAAAAATGAAAACCGATTTT<br>ATCTGAAGGAAGTGAACATCAGCATGTATTTGGTTAATGGCTCCGTTTTCAGCATTGC<br>AAATAACAATCTCAGCTACTGGGATGCCCCCCTGGGAAGTTCTTATATGTGCAACAAA<br>GAGCAGACTGTTTCAGTGTCTGGAGCATTTCAGATAAATACCTTTGATCTAAGGGTTC<br>AGCCTTTCAATGTGACACAAGGAAAGTATTCTACAGCCCAAGAGTGTTCGCTGGATG<br>ATGACACCATTCTAATCCCAATTATAGTTGGTGCTGGTCTTTCAGGCTTGATTATCGTTA<br>TAGTGATTGCTTACGTAATTGGCAGAAGAAAAAGTTATGCTGGATATCAGACTCTG
**AnCar-LaIMT9**	GGAGGAGGAGGAAGCAGAGGAGACGGAGGAGGAGGGAGCAGAGGAGATGGAG<br>GAGGAGGCAGCAGAGGAGACGGGGGAGGAGGAAGTAGAGGAGACGGCGGAGG<br>AGGATCAAGAGGAGACGGCGGGGGAGGAAGCAGGGGAGACGGAGGCGGAGGAA<br>GCCGGGGAGACGGAGGGGGAGGAAGTTTGGAACTTAATTTGACAGATTCAGAAAA<br>TGCCACTTGCCTTTATGCAAAATGGCAGATGAATTTCACAGTACGCTATGAAACTACA<br>AATAAAACTTATAAAACTGTAACCATTTCAGACCATGGCACTGTGACATATAATGGAAG<br>CATTTGTGGGGATGATCAGAATGGTCCCAAAATAGCAGTGCAGTTCGGACCTGGCTT<br>TTCCTGGATTGCGAATTTTACCAAGGCAGCATCTACTTATTCAATTGACAGCGTCTCAT<br>TTTCCTACAACACTGGTGATAACACAACATTTCCTGATGCTGAAGATAAAGGAATTCTT<br>ACTGTTGATGAACTTTTGGCCATCAGAATTCCATTGAATGACCTTTTTAGATGCAATAG<br>TTTATCAACTTTGGAAAAGAATGATGTTGTCCAACACTACTGGGATGTTCTTGTACAA<br>GCTTTTGTCCAAAATGGCACAGTGAGCACAAATGAGTTCCTGTGTGATAAAGACAAA<br>ACTTCAACAGTGGCACCCACCATACACACCACTGTGCCATCTCCTACTACAACACCTAC<br>TCCAAAGGAAAAACCAGAAGCTGGAACCTATTCAGTTAATAATGGCAATGATACTTGT<br>CTGCTGGCTACCATGGGGCTGCAGCTGAACATCACTCAGGATAAGGTTGCTTCAGTT<br>ATTAACATCAACCCCAATACAACTCACTCCACAGGCAGCTGCCGTTCTCACACTGCTCT<br>ACTTAGACTCAATAGCAGCACCATTAAGTATCTAGACTTTGTCTTTGCTGTGAAAAATG<br>AAAACCGATTTTATCTGAAGGAAGTGAACATCAGCATGTATTTGGTTAATGGCTCCGT<br>TTTCAGCATTGCAAATAACAATCTCAGCTACTGGGATGCCCCCCTGGGAAGTTCTTATA<br>TGTGCAACAAAGAGCAGACTGTTTCAGTGTCTGGAGCATTTCAGATAAATACCTTTGA<br>TCTAAGGGTTCAGCCTTTCAATGTGACACAAGGAAAGTATTCTACAGCCCAAGAGTG<br>TTCGCTGGATGATGACACCATTCTAATCCCAATTATAGTTGGTGCTGGTCTTTCAGGCT<br>TGATTATCGTTATAGTGATTGCTTACGTAATTGGCAGAAGAAAAAGTTATGCTGGATAT<br>CAGACTCTG
**AnCar-LaIMT10**	GGAGGAGGAGGAAGCACAAAGCCAAGAGGAGGAGGAGGGAGCACAAAGCCCAG<br>AGGAGGAGGGGGAAGCACAAAACCAAGAGGAGGGGGAGGAAGCACCAAGCCAA<br>GAGGGGGAGGAGGAAGTACAAAGCCAAGGGGAGGAGGAGGCAGCTTGGAACTTA<br>ATTTGACAGATTCAGAAAATGCCACTTGCCTTTATGCAAAATGGCAGATGAATTTCAC<br>AGTACGCTATGAAACTACAAATAAAACTTATAAAACTGTAACCATTTCAGACCATGGC<br>ACTGTGACATATAATGGAAGCATTTGTGGGGATGATCAGAATGGTCCCAAAATAGCA<br>GTGCAGTTCGGACCTGGCTTTTCCTGGATTGCGAATTTTACCAAGGCAGCATCTACTT<br>ATTCAATTGACAGCGTCTCATTTTCCTACAACACTGGTGATAACACAACATTTCCTGAT<br>GCTGAAGATAAAGGAATTCTTACTGTTGATGAACTTTTGGCCATCAGAATTCCATTGA<br>ATGACCTTTTTAGATGCAATAGTTTATCAACTTTGGAAAAGAATGATGTTGTCCAACAC<br>TACTGGGATGTTCTTGTACAAGCTTTTGTCCAAAATGGCACAGTGAGCACAAATGAG<br>TTCCTGTGTGATAAAGACAAAACTTCAACAGTGGCACCCACCATACACACCACTGTGC<br>CATCTCCTACTACAACACCTACTCCAAAGGAAAAACCAGAAGCTGGAACCTATTCAGT<br>TAATAATGGCAATGATACTTGTCTGCTGGCTACCATGGGGCTGCAGCTGAACATCACT

	CAGGATAAGGTTGCTTCAGTTATTAACATCAACCCCAATACAACTCACTCCACAGGCA<br>GCTGCCGTTCTCACACTGCTCTACTTAGACTCAATAGCAGCACCATTAAGTATCTAGAC<br>TTTGTCTTTGCTGTGAAAAATGAAAACCGATTTTATCTGAAGGAAGTGAACATCAGCA<br>TGTATTTGGTTAATGGCTCCGTTTTCAGCATTGCAAATAACAATCTCAGCTACTGGGAT<br>GCCCCCCTGGGAAGTTCTTATATGTGCAACAAAGAGCAGACTGTTTCAGTGTCTGGA<br>GCATTTCAGATAAATACCTTTGATCTAAGGGTTCAGCCTTTCAATGTGACACAAGGAA<br>AGTATTCTACAGCCCAAGAGTGTTCGCTGGATGATGACACCATTCTAATCCCAATTATA<br>GTTGGTGCTGGTCTTTCAGGCTTGATTATCGTTATAGTGATTGCTTACGTAATTGGCAG<br>AAGAAAAAGTTATGCTGGATATCAGACTCTG
**AnCar-LaCTRL**	ATGGTGTGCTTCCGCCTCTTCCCGGTTCCGGGCTCAGGGCTCGTTCTGGTCTGCCTAG<br>TCCTGGGAGCTGTGCGGTCTTATGCAGGAGGAGGAGGATCTTTTCAGCACATGAGC<br>TTTATCGGAGGCGGCGGGAGCTTCCAGCACATGTCATTCATTGGAGGGGGGGGGAG<br>TTTCCAGCACATGAGTTTTATCGGAGGAGGGGGGAGCTTGGAACTTAATTTGACAGA<br>TTCAGAAAATGCCACTTGCCTTTATGCAAAATGGCAGATGAATTTCACAGTACGCTAT<br>GAAACTACAAATAAAACTTATAAAACTGTAACCATTTCAGACCATGGCACTGTGACATA<br>TAATGGAAGCATTTGTGGGGATGATCAGAATGGTCCCAAAATAGCAGTGCAGTTCGG<br>ACCTGGCTTTTCCTGGATTGCGAATTTTACCAAGGCAGCATCTACTTATTCAATTGACA<br>GCGTCTCATTTTCCTACAACACTGGTGATAACACAACATTTCCTGATGCTGAAGATAAA<br>GGAATTCTTACTGTTGATGAACTTTTGGCCATCAGAATTCCATTGAATGACCTTTTTAG<br>ATGCAATAGTTTATCAACTTTGGAAAAGAATGATGTTGTCCAACACTACTGGGATGTT<br>CTTGTACAAGCTTTTGTCCAAAATGGCACAGTGAGCACAAATGAGTTCCTGTGTGATA<br>AAGACAAAACTTCAACAGTGGCACCCACCATACACACCACTGTGCCATCTCCTACTAC<br>AACACCTACTCCAAAGGAAAAACCAGAAGCTGGAACCTATTCAGTTAATAATGGCAA<br>TGATACTTGTCTGCTGGCTACCATGGGGCTGCAGCTGAACATCACTCAGGATAAGGTT<br>GCTTCAGTTATTAACATCAACCCCAATACAACTCACTCCACAGGCAGCTGCCGTTCTC<br>ACACTGCTCTACTTAGACTCAATAGCAGCACCATTAAGTATCTAGACTTTGTCTTTGCT<br>GTGAAAAATGAAAACCGATTTTATCTGAAGGAAGTGAACATCAGCATGTATTTGGTTA<br>ATGGCTCCGTTTTCAGCATTGCAAATAACAATCTCAGCTACTGGGATGCCCCCCTGGG<br>AAGTTCTTATATGTGCAACAAAGAGCAGACTGTTTCAGTGTCTGGAGCATTTCAGATA<br>AATACCTTTGATCTAAGGGTTCAGCCTTTCAATGTGACACAAGGAAAGTATTCTACAG<br>CCCAAGAGTGTTCGCTGGATGATGACACCATTCTAATCCCAATTATAGTTGGTGCTGG<br>TCTTTCAGGCTTGATTATCGTTATAGTGATTGCTTACGTAATTGGCAGAAGAAAAAGTT<br>ATGCTGGATATCAGACTCTG

Supplementary Materials Excel 1. Certain proteins were identified containing the sequence of LPSSGAA

Movie S1. Two-photon microscopy showed that macrophages expressing tdTomato protein ingested exosome labelled by DID dye in a dynamic real-time way from 0 min to 120 min.

Movie S2. The abnormality of gait in right leg in *HIF-1α*$^{flox/flox}$ mice compared with *HIF-1α*$^{flox/flox}$ *–LysM*^{cre} mice.
